# The Brain's Router: A Cortical Network Model of Serial Processing in the Primate Brain

**DOI:** 10.1371/journal.pcbi.1000765

**Published:** 2010-04-29

**Authors:** Ariel Zylberberg, Diego Fernández Slezak, Pieter R. Roelfsema, Stanislas Dehaene, Mariano Sigman

**Affiliations:** 1 Laboratory of Integrative Neuroscience, Physics Department, University of Buenos Aires, Buenos Aires, Argentina; 2 Institute of Biomedical Engineering, Faculty of Engineering, University of Buenos Aires, Buenos Aires, Argentina; 3 Laboratory of Complex Systems, Computer Science Department, University of Buenos Aires, Buenos Aires, Argentina; 4 Netherlands Institute for Neuroscience, An Institute of the Royal Netherlands Academy of Arts and Sciences (KNAW), Amsterdam, The Netherlands; 5 Department of Integrative Neurophysiology, Vrije Universiteit, Amsterdam, The Netherlands; 6 INSERM, CEA, Cognitive Neuroimaging Unit, Orsay, France; 7 Collège de France, Paris, France; University College London, United Kingdom

## Abstract

The human brain efficiently solves certain operations such as object recognition and categorization through a massively parallel network of dedicated processors. However, human cognition also relies on the ability to perform an arbitrarily large set of tasks by flexibly recombining different processors into a novel chain. This flexibility comes at the cost of a severe slowing down and a seriality of operations (100–500 ms per step). A limit on parallel processing is demonstrated in experimental setups such as the psychological refractory period (PRP) and the attentional blink (AB) in which the processing of an element either significantly delays (PRP) or impedes conscious access (AB) of a second, rapidly presented element. Here we present a spiking-neuron implementation of a cognitive architecture where a large number of local parallel processors assemble together to produce goal-driven behavior. The precise mapping of incoming sensory stimuli onto motor representations relies on a “router” network capable of flexibly interconnecting processors and rapidly changing its configuration from one task to another. Simulations show that, when presented with dual-task stimuli, the network exhibits parallel processing at peripheral sensory levels, a memory buffer capable of keeping the result of sensory processing on hold, and a slow serial performance at the router stage, resulting in a performance bottleneck. The network captures the detailed dynamics of human behavior during dual-task-performance, including both mean RTs and RT distributions, and establishes concrete predictions on neuronal dynamics during dual-task experiments in humans and non-human primates.

## Author Summary

A ubiquitous aspect of brain function is its quasi-modular and massively parallel organization. The paradox is that this extraordinary parallel machine is incapable of performing a single large arithmetic calculation. How come it is so easy to recognize moving objects, but so difficult to multiply 357 times 289? And why, if we can simultaneously coordinate walking, group contours, segment surfaces, talk and listen to noisy speech, can we only make one decision at a time? Here we explored the emergence of serial processing in the primate brain. We developed a spiking-neuron implementation of a cognitive architecture in which the precise sensory-motor mapping relies on a network capable of flexibly interconnecting processors and rapidly changing its configuration from one task to another. Simulations show that, when presented with dual-task stimuli, the network exhibits parallel processing at peripheral sensory levels, a memory buffer capable of keeping the result of sensory processing on hold. However, control routing mechanisms result in serial performance at the router stage. Our results suggest that seriality in dual (or multiple) task performance results as a consequence of inhibition within the control networks needed for precise “routing” of information flow across a vast number of possible task configurations.

## Introduction

A ubiquitous aspect of brain function is its modular organization, with a large number of processors (neurons, columns, or entire areas) operating simultaneously and in parallel. Human cognition relies, to a large extent, on the ability to perform an arbitrarily large set of tasks by flexibly recombining different processors into a novel chain (e.g. respond with the right hand to the red square) [1]–[3]. Yet this flexibility does not happen without a cost. Chaining individual computations is done at a very slow pace (100–500 ms per step) and with a considerable temporary tying-up of the brain's resources, generating what is known as “dual-task interference” – the inability to perform several tasks at once [4]–[8]. Several cognitive theories support this view, arguing that while most mental operations are modular and parallel, certain specific processes which establish flexible links amongst existing processors impose a serial processing bottleneck [3], [9]–[15].

The psychological refractory period (PRP) provides a classic and clear demonstration in experimental psychology of the coexistence of parallel processing and serial processing bottlenecks within a cognitive task. When performing two tasks in rapid succession on two successively presented targets T1 and T2, delays are observed in some but not all of the T2 processing stages. Analysis of these delays suggests that a “central decision stage” suffers from seriality while perceptual and response operations occur in parallel [4], [6], [7], [16], [17]. Despite the fact that the PRP has been one the most widely studied paradigms to investigate dual-task interference, no network implementation had been proposed which provides a plausible implementation of its underlying mechanisms. Boxological and schematical models of the PRP [4], [18], [19] have successfully determined a theoretical framework which provides a synthesis of two basic aspects of cognitive architecture: 1) its chronometric organization, 2) its components that can act in parallel and those that impose seriality. According to these models, each task involves three successive stages of processing: a perceptual, a central, and a motor component. The perceptual stage of sensory processing - which is performed in a modular (parallel) fashion - does not provide a major contribution to temporal variability. A subsequent stage of serial processing involves a stochastic integration process, traditionally used to model decision making in single tasks [20]–[23] and is a main source for the variability in response time. In contrast, the last motor processing stage has only a small contribution to response variability and can be performed in parallel without interfering with other processing stages from concurrent tasks. Despite their simplicity, these models have been very successful in explaining a broad range of behavioral data, including the complex response time distributions of dual-task experiments, which can be precisely predicted only after untangling the serial and parallel stages of each task [18].

Until now, the modeling of dual tasks is only specified at a level of mathematical description and functional cognitive architecture [4], [18], [24], [25]. At the neurophysiological level, understanding what kind of collective neural organization leads from massively parallel single-unit processing to a serial unfolding of two successive decisions has not been established. This situation is, to a large degree, due to the fact that there have been detailed monkey electrophysiology of single-task decision making [26], [27], but no comparable investigation of dual-tasks. Here we present an effort to bridge this gap between an abstract mathematical description and the underlying complex neurophysiology. We present a detailed model, based on realistic properties of spiking neurons which is capable of flexibly linking processors to form novel tasks. As a consequence of this flexibility, the network exhibits a functional serial bottleneck at the level of the “router” circuit needed to link processors. The model presents detailed predictions for future electrophysiological studies of dual-tasks and serial computations in the human and non-human primate brain.

## Results

### Architecture of the Model

In accordance with previous theoretical proposals [28], [29] here we propose that seriality in dual (or multiple) task performance results as a consequence of inhibition within the control networks needed for precise “routing” of information flow across a vast, virtually infinite, number of possible task configurations. To examine this hypothesis, we will explore dual-task performance in a recurrent network of spiking neurons capable of performing flexible routing of information according to specific task instructions. Contrary to previous computational work addressing flexible mapping [30]–[33], our objective is not to study flexible behavior *per se* but to understand the conditions under which a computational model capable of flexible sensory-motor mapping shows patterns of interference when two tasks have to be performed simultaneously or in close succession [17], [18], [34].

**Figure 1:**
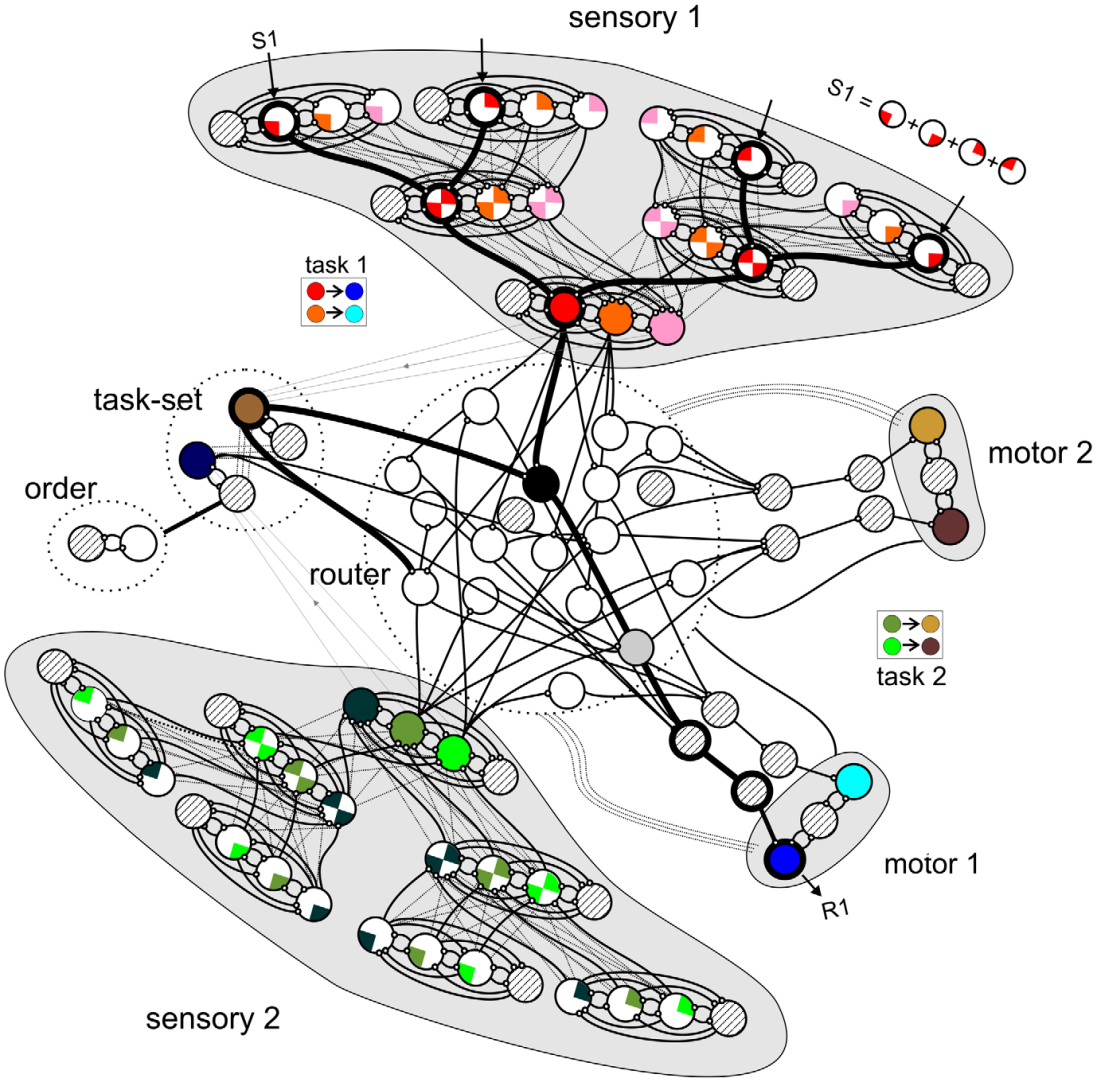
Network architecture. Schematic of the spiking neuron network model. Each population, represented with a circle, contains between 80 and 640 neurons. Circles with diagonal textures indicate inhibitory populations and all other circles indicate populations of pyramidal cells. Whenever two populations of neurons are connected this indicates full connectivity between them. The network includes two sensory modalities (sensory 1 and 2), organized in a hierarchy in which each successive layer receives inputs – mediated by rapid (time constant of 2ms) AMPA receptors - from various populations of the previous layer thus generating progressively more complex receptive fields. Each stimulus (for example, S1) is represented by the co-activation of four specific neural populations in the first layer of the sensory hierarchy. Just for illustration purposes, each stimulus is represented as a solid circle and the different features of this stimulus as parts of this circle, i.e. the 4 red neurons in the first layer represent a stimulus when they are active together. Sensory modules are also connected through non-specific feedback connections mediated by slow (time constant of 100ms) NMDA receptors. Both sensory modalities converge to the router, which is a common integrator. The integrator neurons feed back to the sensory neurons, generating recurrent activity which can maintain and amplify sensory information. Integrator neurons connect to response neurons and thus route information from sensory to motor neurons. Subsets of the neurons in the router link information from stimuli to responses in a flexible manner. Router neurons also receive input from task-setting neurons and thus act as detectors of the conjunction of the relevant task and the appropriate stimulus. The circuit involved in mapping S1 to R1 of Task 1 as well as the task-setting of Task 1 is emphasized in bold. Response execution is triggered by a set of bursting neurons that signal a threshold-cross of the input received from the routing neurons that integrate information. Response neurons feed back to the router and to inhibit the neurons immediately after the response. This inhibition prevents perseveration and is required to stabilize the network in a single response mode. In a typical PRP experiment, which we model here, subjects are instructed to respond to both tasks as fast as possible in a particular order. To enforce this response order in the network we organized the task-setting neurons in a hierarchy [52] in which the neurons coding for Task 1 and Task 2 are controlled by a switch composed of task-order units (see Materials and Methods section for a detailed description).

Following classic experimental procedures of the PRP [35], the interference experiments we address here involve different sensory modalities, to avoid sources of interference in early sensory processing (with the exception of the last section, where we investigate the effects of masking). The model that we simulate is described in detail in the Materials and Methods section and in Figure 1. It includes two sensory modalities organized in a hierarchy in which each successive layer receives inputs from neurons of the previous layer thus generating progressively complex receptive fields. Within each hierarchical level, for simplicity we explore in detail only two distinct neural populations for each sensory modality, which correspond to the neural coding of the two task-relevant dimensions (red and orange populations in Figure 1 representing, for example, a high and low pitch sound, respectively). Other task-irrelevant stimuli were encoded by a large pool of non task-selective excitatory neurons (pink populations in Figure 1), as done in many other spiking networks modeling decision-making [36].

Each element in this sensory hierarchy is a canonical cortical circuit comprising excitatory pyramidal cells and local inhibitory cells, previously shown to be capable of performing elementary functions of working memory and decision making [36]–[38]. Only excitatory pyramidal cells project with long-range connections to neurons higher and lower in the sensory hierarchy, while inhibitory neurons only project locally. Feedforward and feedback connections in the model differ both in the properties of the receptors that mediate the transmission as well as in their specificity [39]–[42]. Feedforward connections are highly specific: Each neuron projects to a single homogeneous population in the next higher level. For simplicity, they are assumed to be all mediated by fast AMPA receptors, although in reality a small fraction of NMDA receptors would be expected. In the reciprocal direction, feedback connections are more broadly connected: each neuron sends non-specific feedback connections to all excitatory cells in the previous level [40], [41]. Again, for simplicity we assume that feedback transmission is mediated by slow NMDA receptors. Since the contribution of NMDA receptors to synaptic transmission varies with the level of postsynaptic depolarization, this ordering of glutamate receptors between the feedforward and feedback streams broadly assigns a driving role to the feedforward input and a modulatory one to the feedback, as in previous models [43].

Both sensory modalities project to a router which connects the sensory representations to a set of possible responses. Neurons in the router integrate sensory evidence and trigger a response when their activity reaches a threshold [44].

An explicit instruction - presented before the stimulus – sets the task for a given trial, i.e. specifies the specific mapping which indicates which response has to be executed when the stimulus is presented. The network that stores task instructions is referred throughout this work as the *task-setting network*. Excitatory populations in this network are activated by the presence of task-relevant stimuli in sensory areas and, through their patterns of projection to “router” neurons (see below), encode different stimulus-response mappings. As with the sensory modalities, we only simulate two task-setting populations which are sufficient for the experiments considered here.

An important aspect of our model is a circuit which we refer as the “router”. As in previous models of flexible decision making that do not rely on synaptic plasticity to dynamically adjust their behavior [33], [45], [46], task-setting neurons affect the decision process by gating a specific subset of “router” neurons, which implement the possible mappings between stimuli and responses. Here we assume a reduced ensemble of stimuli and responses and simply model as many selective populations in the router as there are combinations of stimuli and responses [33], [47]. Simulating a completely flexible network capable of mapping arbitrarily large stimulus and response sets, would require a high degree of overlap in the cortical representation implemented by task-setting and routing neurons. We will come back to this possibility and its possible implications for serial processing in the discussion.

**Figure 2:**
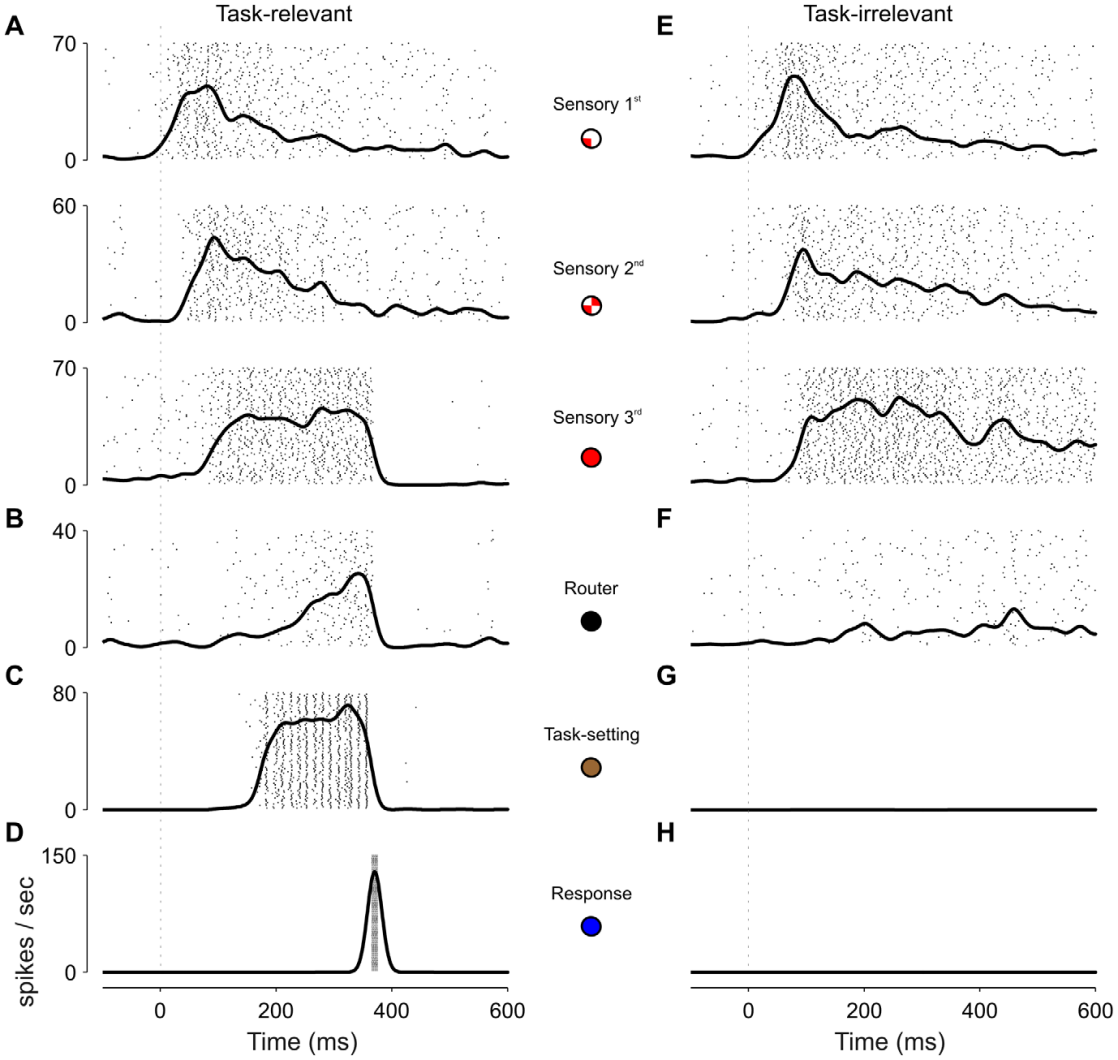
Single-trial dynamics for task relevant and irrelevant stimuli. Firing rates of representative trials of task-relevant (A–D) and task-irrelevant (E–H) stimuli. Each panel shows the firing rates averaged across a population (thick line) overlapped with spike rasters (each row of dots represent the spiking activity of a neuron in the population). Average firing rates were calculated by convolving the spike raster from a single trial with a gaussian filter of σ = 12*ms*. (A) Stimulus presentation (indicated with a dashed vertical line) generates a wave of activity that propagates through the successive stages of the sensory hierarchy. The colored circles represent the features coded by the various populations, following the notation of figure 1. (B) Router neurons show ramping activity until a response threshold is reached. (C) Activity in task-setting neurons is triggered by excitatory input from sensory neurons and is sustained for the duration of the task. (D) The response is signaled by a burst of excitatory neurons in the response network. (E–H) Same as panels A–D, but with the connections from sensory to task-setting areas removed. In the absence of projection from task-setting neurons (G) the activity in the router (F) does not reach the threshold to trigger a response in motor areas (H), despite strong activation in sensory areas (E).

As with all other neurons in the network, task-setting neurons are entailed with self excitation and lateral inhibition. Excitatory neurons in the task-setting network are connected to the router through NMDA connections. When an excitatory population of the task-setting network is in an “active” state it excites the subset of neurons in the router receiving inputs from task relevant sensory populations and connecting them to the appropriate motor populations. A neuron in the router which receives excitation from task-setting neurons is set in a mode of integration in which it can accumulate sensory information (Text S1,A). This architecture also serves as a selection mechanism, assuring that task-irrelevant stimuli that are represented in sensory cortex do not elicit any output (Figure 2).

Response execution is triggered in response selection networks (motor 1 and 2 in Figure 1) by a set of bursting neurons that signal a threshold-crossing of the input received from the integrating neurons, modeled as in previous work by Wang and collaborators [44].

To ensure that the network did not enter in a response perseveration mode (Figure S1), we implemented an inhibition of return mechanism [48] typical of a control network. After response execution, response neurons feed back to inhibit the sensory, routing and task-setting neurons involved in the task (similar to the “termination” signals in Dehaene and Changeux, 1997 [49] and recently observed in single-cell recordings in awake behaving monkeys performing a sequential task [50]).

This architecture ensured that the network did not respond spontaneously, to irrelevant stimuli or to mappings different than those set by the explicit task-instruction and that it did not show perseveration of responses to task-relevant stimuli. We emphasize that here we have not investigated how a large repertoire of tasks can be encoded with a finite number of neurons. Rather, we ensure that the network has stable performance for a small number of tasks and then explore the operation of this network during dual-task performance.

Our simulations of dual task experiments showed that when both tasks were close together in time, response order could be reversed on a fraction of trials so that the first response was given to the stimulus that was presented second (Figure S2). This coincides with experimental observation in task-interference experiments when the response order is not fixed [51]. Here we wanted to explore a comparatively simpler situation, typically studied in psychophysical experiments, in which participants are explicitly told to respond to two tasks in a specific order, as fast as possible. This required the implementation of a task-setting network [52] that determined the order of the tasks. The task-setting network was bistable. It was composed of two excitatory populations that projected to the inhibitory population of the other task. Three hundred milliseconds before the presentation of the first stimulus, excitatory neurons in the order-setting network are activated by a brief (100 ms) external input. Due to the strong self-recurrent connections, the network maintains high levels of activity after removal of the external input and tonically inhibits T2 neurons in the task-setting network. When the response to T1 is emitted, inhibition from the router resets the order-network permitting the activation of T2 task setting-neurons (Text S1,B).

In summary, we generated a network based on a large-scale implementation of simple canonical neuronal circuits endowed with self-recurrence and lateral inhibition. The network has a hierarchical sensory organization which ultimately feeds stochastic evidence to “router” neurons which (if activated by a specific task-setting context) both accumulate evidence towards a motor decision and route sensory input to the relevant motor neurons.

### Time Course of Neural Activations during Single-Task Performance

Each stimulus has four features. The four populations encoding low-level features of a stimulus receive a brief pulse of constant current during stimulus presentation (100 ms). This initial impulse generates a transient response in the earliest input neurons (Figure 2A–D), which increase their firing rate from the default level of around 2 Hz to around 40 Hz. This transient response initiates a wave of activation that propagates through the network [47], [53], [54]. Each layer works as an integrator of the previous layer and thus the neural response becomes increasingly expanded in time as one progress in the hierarchy. At the highest level, recurrent connections are strong enough to assure a very low decay rate of stimulus information, resulting in an effective form of working memory as observed in several areas of occipito-temporal and frontal cortex [55]–[57].

The last stage in the sensory hierarchy projects to the router using AMPA receptors. Neurons in the router also receive currents from task-setting neurons, but these projections use NMDA receptors. These NMDA currents control the recurrence in the router, and they determine the degree of integration of AMPA currents. As a result of this architecture, neurons in the router act as detectors of the conjunction of stimulus presence and task relevance as observed in [58]–[60]. A neuron which receives task-setting currents integrates the sensory input rapidly (Figure 2B), while a neuron that does not integrates the input only partially (Figure 2E–H). Thus, task-setting neurons accomplish their role by assuring that the wave in the sensory system initiated by an irrelevant stimulus does not trigger a response. The integration process continues until a threshold is crossed, which is signaled by a nonlinear response: a powerful burst of spikes in the motor network (Figure 2D). The activation of these response neurons, in turn, initiates a cascade of feed-back inhibition that resets activation in task-related neurons [50].

### Time Course of Neural Activations during Dual-Task Performance

The principal aim of this paper is to explore the operation of the model in a classic dual-task paradigm: the psychologically refractory period (PRP), widely studied in the psychophysical literature. We explored the response of the model with two different stimuli, presented simultaneously or at a short stimulus onset asynchrony (SOA). When the separation between stimuli (SOA) is much longer than the response time to the first task (RT1), the neural activations associated with the first and second task do not interfere with each other and the observed dynamics is similar to that observed during single-task performance (Figure 2A–D).

**Figure 3:**
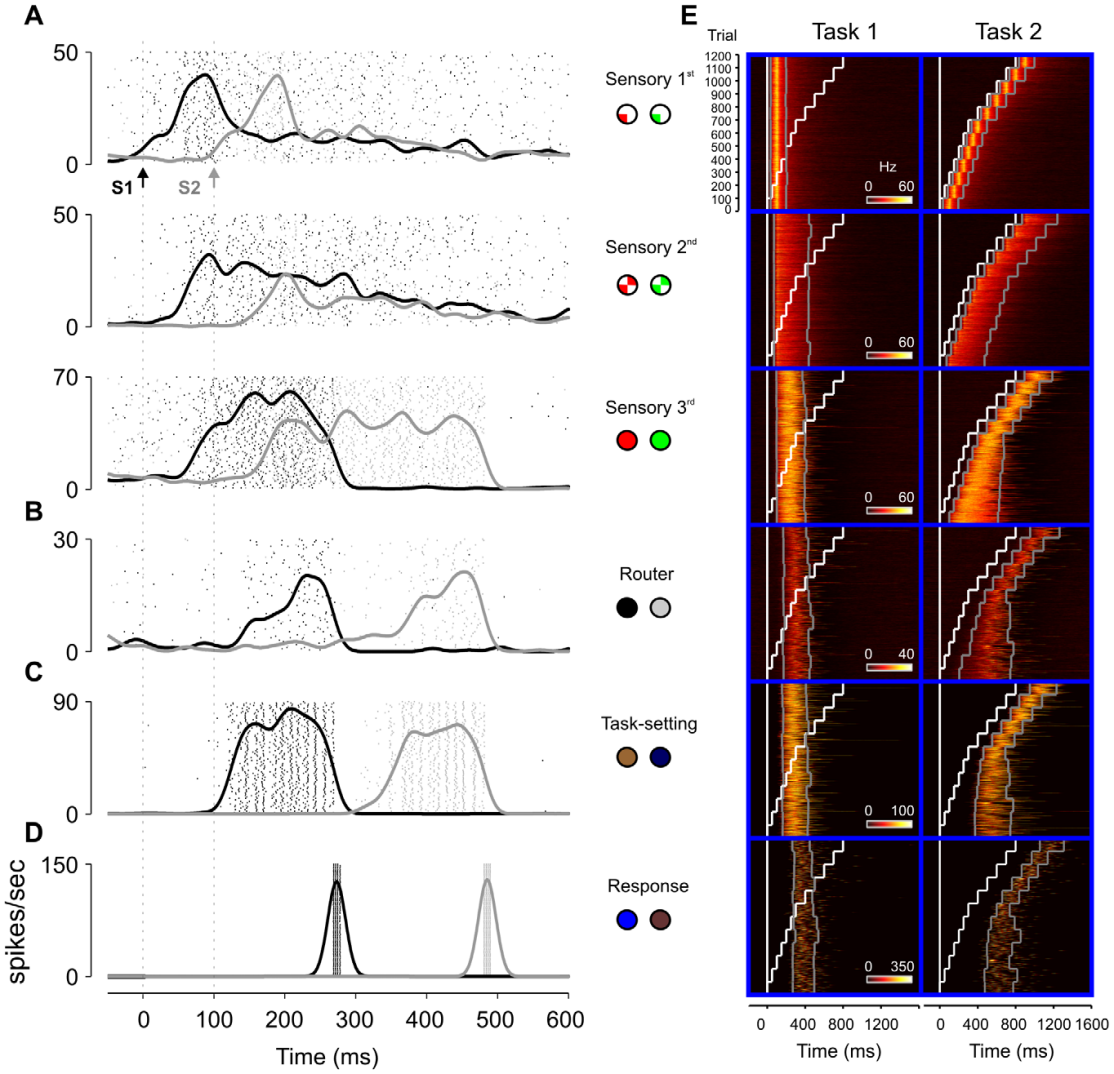
Neural activations during dual-task performance. (A–D) Firing rates in the dual-task condition inside the interference regime (SOA = 100ms). Each panel is defined as in Figure 2, with black and grey lines corresponding to populations from the first and second tasks, respectively. The specific populations plotted are indicated with colored circles, following the notation of Figure 1 (the circles to the left correspond to the first task). (E) Firing rates are plotted for 1200 trials (100 at each SOA) for neurons responding to the first and second tasks (left and right columns in each panel, respectively). White lines indicate the onset of the stimuli of task 1 and 2, and grey lines mark the specific times at which the average activity at each SOA crossed 1/3 of its peak value. In early sensory areas, both the onset and offset of the response are time-locked to stimulus presentation at short SOA, thus indicating a completely parallel mode of activation. In contrast, task-setting, response and integrating neurons show a highly serial activation profile. The onset and the offset of these neurons for task 2 in the interference regime are locked to the end of the corresponding process of task 1. In the non-interference regime, however, the onset of these neurons is locked to stimulus presentation. Higher sensory modules showed a hybrid profile, indicating that the same neuron can be involved in a phasic parallel response and also exhibits sustained activity until the response. In the interference regime, the onset of these neurons is locked to the presentation of the stimulus, but the offset show a sequential locking to the ending of task 1. Firing rates were calculated by filtering the instantaneous population firing rate with an exponential causal kernel with a time constant of 20 ms.

The most interesting situation is for SOA values close to or shorter than RT1 (Figure 3A–D, SOA = 100ms) in which case the two waves of activation evoked by each stimulus partially interfere. In the model, this interference does not occur at the sensory level: even at short SOA, while a first target T1 is being processed, sensory neurons associated with the second target T2 still initiate a wave of activations which is very similar to that in the single-task condition. However, due to competition between task-setting neurons, the routing neurons of T2 are not gated and hence do not integrate sensory information while T1 is being processed. In this instance there is a very interesting dissociation: local-recurrence in the sensory hierarchy is sufficient to maintain T2 stimulus information, but this information is not piped to the motor response and awaits liberation of the router. This constitutes a key aspect of this network – during a temporary waiting period, T2 has to be maintained in a “local memory” which does not propagate throughout the network. After the response to the first task has been executed, the T1 pathway is reset and Task 2 setting neurons activate, gating the router neurons of T2 and allowing them to begin to integrate information about the second incoming stimulus. Thus, the shift in the locus of “task-related attention” (which information is amplified in sensory areas and routed to response networks) is the natural consequence of the progression of the task in the router and task-setting network.

Note that the second key aspect of our network is that routing neurons of T1 and T2 cannot be simultaneously activated. In our network this is controlled through a competition between task setting neurons, but a similar result would be obtained if this competition would be implemented by lateral inhibition between routing neurons. This would occur, for example, if the number of possible mappings largely exceeds the number of neurons in the router so that routing can only occur by a distributed assembly of active cells. We will come back to this possibility in the discussion.

In the interference regime, the network includes groups of neurons with very different response properties (Figure 3E); the existence of these different types of neuronal firing patterns constitutes a key prediction of our simulations. Early sensory neurons show a response which is essentially unaffected by interference, reflecting fully parallel behavior. In contrast, the motor and task-setting neurons are strictly serial, only showing strong activation after task 1 has been completed. The behavior of the router neurons is intermediate; they are mostly serial, but can undergo moderate integration (insufficient to boost a response) before completion of T1. Interestingly, late sensory neurons act as a buffer. They have an onset which is locked to the stimulus and are active until the response, so that they hold a memory of T2 which is retrieved when the router becomes available. This population of neurons is therefore engaged in different components of the task; first, a transient response which results in stimulus encoding, and second, a later memory trace which is eventually broadcasted to the motor neurons involved in the second task.

All the previous analysis relied on spiking activity. Recently, much effort has been devoted to understand the relevance of complementary measures of brain function such as synaptic currents, local field potentials, and induced oscillations. Our neuronal network has the potential to study these measures.

We first explored whether input currents in the router could be more informative than spiking activity of T2 processing stages. We measured input currents to the router at different processing stages of T2: Spontaneous activity, S2 queuing (memory phase), and S2 routing. During queuing, currents in the router reflected a steady level of activity which was significantly larger than during spontaneous activity (Figure S3). Thus, during this regime, subthreshold activity in the router is tightly coupled to spiking activity of late sensory neurons. During the routing stage, synaptic current activity ramps, coupling to the progression of spiking activity in the router. An interesting observation was that this pattern was virtually identical for all receptor currents (NMDA, AMPA and GABA). Although the input from the task-setting network is carried by NMDA-receptors, the local amplification in the router circuit also engages AMPA currents and the NMDA specificity is lost very rapidly (Figure S3).

The task-switching circuit was endowed with high efficiency inhibition to achieve rapid switching from one task-setting program to another. This endowed the task-setting circuit with high frequency oscillations as can be seen in the raster plots of Figure 2. Since the task-setting circuit drives the router, we asked how these oscillations propagate into the network and whether measures of oscillatory activity could be more informative than simply spiking activity to identify distinct processing stages from neuronal responses. We analyzed the spectrogram of sensory, routing and task setting T2 neurons throughout the trial (Figure S4). Responses were locked to RT1. Both router and task setting neurons showed clear event-related spectrograms, as seen for firing rates. The spectral content of the responses of both populations are quite distinct: task-setting circuit activity occurs in high-frequency bands (peaking around 70 Hz) while router neurons, which act as slow integrators, display low-frequency responses (∼20 Hz). Router neurons do not inherit high frequency oscillations of the driving task-setting neurons because these connections are mostly mediated through NMDA receptors which have a slow time constant.

Rhythmic activity in the sensory neurons showed distinct oscillatory activity during buffering and routing (Figure S4, left panel). During routing, responses of sensory neurons showed high power in the 40–60 Hz range while during routing they were more broad band and showed an increase in lower-frequency activity. Firing rates of sensory neurons during buffering and routing were not different (Figure 2). Spike density coherence between sensory and router neurons also showed distinct profiles during distinct phases of task processing: phase coherence was not-significant during spontaneous activity, it showed significant coupling for low frequencies during routing and broad-band coherence during T2 queuing (Figure S5).

### Response Times in Dual-Task Performance

**Figure 4:**
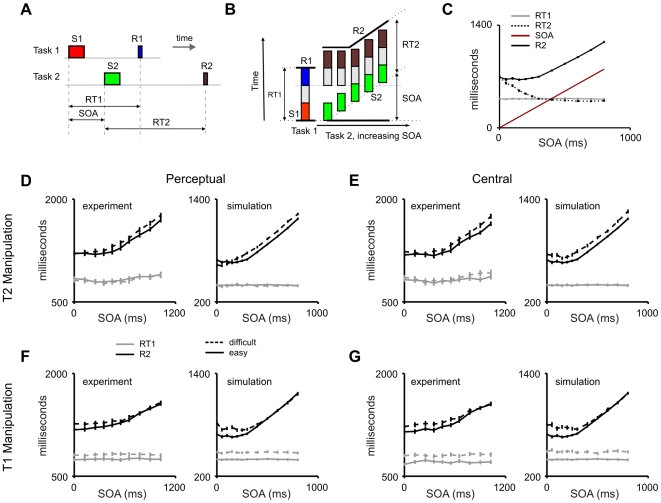
Mean response times: fingerprints of dual-task interference. (A) Sketch of the PRP paradigm. Stimulus S1 is mapped to R1, and stimulus S2 to response R2. RT1 is defined as the time between S1 onset and the response R1. RT2 is defined as the time between the onset of S2 and the response R2. The SOA - defined as the time between onsets of S1 and S2 - is systematically varied, typically between 0 and 1000 ms. (B) Scheme of the mathematical formalism traditionally used to explain the delay in RT2 during the PRP. The vertical axis labels RT. The column on the left indicates the first task, and each colored box within the column represents a different stage of processing: Perceptual component (red), Central component (grey), and Motor component (blue). The series of columns on the right indicate the processing time for task 2 at different SOA, labeled on the x-axis. For each column, the three different boxes represent the three different stages of task 2: Perceptual component (green), Central component (grey), and Motor component (brown). As SOA progresses, the Perceptual component starts later. All components can be performed in parallel except for the Central component, which establishes a bottleneck. (C) Effect of SOA manipulations in response times for the proposed neural architecture. Average response times to the second task show a dependency on SOA similar to observations from PRP experiments: RT2 decreased with SOA within the interference range with a slope of −1, and is constant in the non-interference regime. RT1 is unaffected by SOA manipulations. In most PRP studies, response times are measured from the onset of the corresponding stimulus (T1 or T2). Other studies have used a different convention in which response times to both tasks are reported from trial onset (i.e., onset of T1). Here we show the PRP effect under both conventions, by defining the variable R2 = RT2 + SOA. The PRP effect is observed as an invariance of R2 with SOA for short SOA values, and a linear increase of R2 with SOA for large SOA values. Data points show averages across 300 trials. Error bars depict the standard error of the mean. (D–G) Effect of task complexity and SOA in response times. Each panel (containing two plots) defines the manipulation type (perceptual or central) and the affected task. Human data (taken from [18]) is shown to the left in each panel. To maintain the convention adopted in the experimental study [18], response times are shown relative to the onset of the first task. In each plot both easy (without manipulation, solid line) and difficult (with manipulation, dashed line) conditions are shown. RT1 is shown in grey, and R2 is shown in black. (D,F) We first varied the response complexity of the stimulus, changing the layer of the sensory hierarchy which feeds the integrator (Perceptual). This effect resulted in an increase of (RT1) when this factor affected the first task (F) which propagated to the second task (increase in RT2) within the interference regime. When the factor affected the second task (D) we observed no change in the first task, and a change in RT2 only outside of the interference regime, indicating that this manipulation can be absorbed during the PRP. This is exactly what is expected in the classic PRP model from a ‘pre-bottleneck’ manipulation [4]. (E,G) We also varied the stimulus ambiguity (i.e. the relative input currents to each of the two competing sensory populations) (Central). When the ambiguity of the first task was increased (G), we observed an increase of (RT1) which propagated to the second task (increase in RT2) within the interference regime. When the factor affected the second task (E) we observed an effect on RT2 both inside and outside the interference regime. This is exactly what is expected in the classic PRP model from a ‘bottleneck’ manipulation [4]. Data points show averages across 200 trials, except the baseline data (easy condition) that were averaged across 300 trials.

An appealing aspect of the PRP paradigm (Figure 4A–B) is that it is associated with a large number of chronometric observations. We explored whether the network shows a behavior in accordance with these observations including the dependence of mean RT (and RT distributions) with SOA and the differential effects of pre and post-bottleneck manipulations.

Specifically, the main experimental characteristics of the PRP phenomenon are [18], [34], [35]:

1. RT2 shows a linear decrease with slope of −1 for short SOA and a slope of 0 for large SOA
2. RT1 is typically unaffected by SOA
3. Pre-bottleneck manipulations (experimental factors that affect sensory processing) additively affect both RT1 and RT2 inside the interference range when the first task is being manipulated. When the second task is manipulated, under-additive effects are seen at short SOA, due to the absorption of pre-bottleneck components while T2 is being queued by T1 processing
4. Bottleneck manipulations (experimental factors that affect the difficulty of the S-R mapping) additively affect the task that is being manipulated
5. RT distributions are long-tailed (Wald-type distributions)
6. RT2 tightly covaries with RT1, but only for short SOA (i.e. in the interference regime)
7. RT2 variance increases as SOA decreases, since it accumulates the variability of both RT1 and RT2 in the interference regime

**Figure 5:**
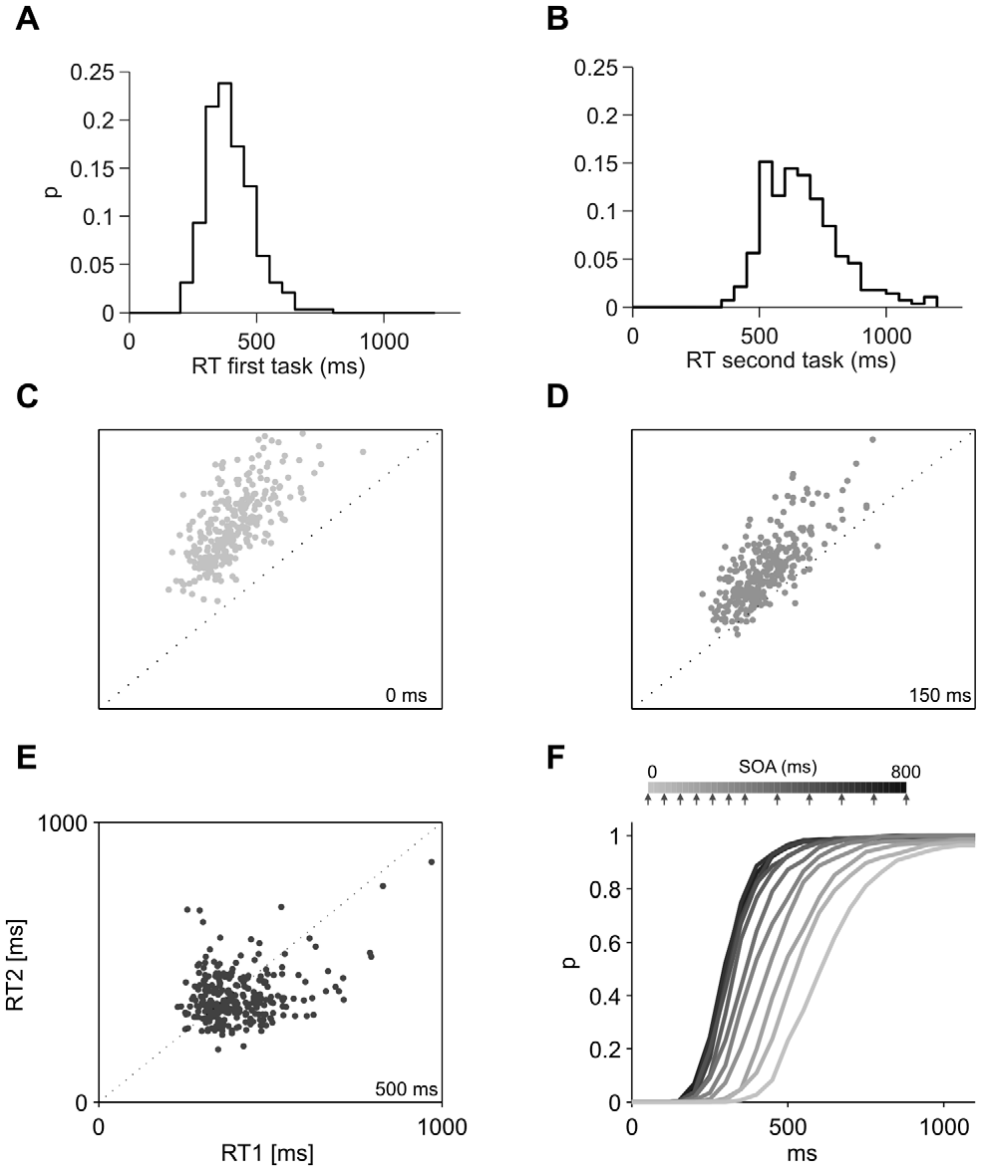
Response time distributions in dual-task execution. (A and B) The model produces distributions of response times with a long tail. (B) As observed experimentally, for short SOA values (SOA = 0 ms in the figure), RT2 is more variable, since it concatenates the variances of both tasks. (C to E) Scatter plot of RT2 vs. RT1 for SOAs of 0ms (C), 150ms (D) and 500ms (E) (300 trials for each SOA). For short SOA values, the RTs are tightly correlated, a correlation that is caused by interference and sequentiality. (F) Cumulative RT distribution for varying SOA values. For increasing SOA values both the mean and the variance decrease. Simulated SOA values are indicated in the legend with arrows.

We first explored the main effects of the PRP (without specific task manipulations) by simulating an experiment in which two stimuli were presented at an SOA which varied between 0 and 800 ms, sampled at [0, 50, 100, 150, 200, 250, 300, 400, 500, 600, 700, 800] ms (Figure 4C). Response times were defined as the time interval between the onset of the stimulus signaling each task and the peak of the motor burst. The network virtually made no mistakes (error rates were less than 0.1% for both tasks), which was expected given that the two different stimuli have non-overlapping representations in each sensory modality. We observed that the network behavior captured all the predictions listed above (Figures 4 and 5). RT1 was unaffected by SOA (Figure 4C–G). Although, the presentation of the second stimulus provides input to the task-setting neurons of T2, this network is configured in a winner-take-all mode and the top-down control of T1 over the router neurons is virtually unaffected by the incoming stimuli. Thus, S2 was never strong enough to overwrite T1 in the task setting network as long as this task was ongoing.

Second, we observed the classic RT2 profile with varying SOA values: An initial decrease with a slope of −1 (Figure 4C). This indicates that T2 completion is strictly serial even though some aspects of T2 processing are carried out in parallel with T1 (Figure 3). As SOA increased and reached the average value of RT1, the two tasks became increasingly independent. The stochasticity of the system (see below for an analysis of RT distributions) assured that this elbow –i.e. the regime in which RT2 becomes independent of SOA was not sharp and thus RT2 showed a curved decay which reached a horizontal asymptote after about 300 ms, as observed in human psychophysics (Figure 4C).

Based on typical experimental procedures, we then explored the effect of different manipulations on the first and second task on mean response times, and their interaction with SOA (Figure 4D–G).

First we investigated the effect of changing the complexity of sensory processing. In a number comparison task, changing the notation (for instance replacing the digit 3 by the word *three*) results in an increase in response time which is absorbed during the PRP (i.e., more elaborate sensory processing of S2 can occur while central processing for T2 is blocked by the processing of task 1, therefore not increasing RT2 at short SOA) [18]. A simple model of word recognition predicts that complex combinations of characters are encoded in successive layers of a feed-forward scheme [53], [61]. To model this experimental factor in our network, we simply added an additional processing level in the sensory hierarchy. We first applied this manipulation to task 1, and observed an additive effect on RT1, which did not depend on the SOA (Figure 4F). This effect propagated to RT2 in the interference regime. This shows that the network functions strictly in a first-come first-served basis. Manipulating the second task affected RT2 for long SOA values, but had no effect at short SOA (Figure 4D), indicating that the additional sensory processing can be carried out in parallel with T1 processing. This absorption of pre-bottleneck manipulations constitutes one of the critical predictions of theoretical models of the PRP (Text S1,C).

We then explored another important manipulation which affects the complexity of the sensory-motor mapping, i.e. the amount of sensory evidence in favor of the correct decision. In experiments in which a decision is taken on an analog variable (movement, intensity, numerosity, size etc…) the two competing stimuli can be made arbitrarily close, rendering the decision progressively more difficult. This results in increased errors and RTs, and attractor dynamic networks have been very successful in modeling these phenomena [37], [62]. This *distance* manipulation in a PRP setup results in a bottleneck manipulation which is not absorbed in the PRP. Here, as conventionally done, we modulated the amount of evidence by changing the relative input currents of each of the two competing sensory populations (Figure 4E,G). We applied this manipulation to the first task, and observed an increase in RT1 unaffected by SOA (Figure 4G). This effect propagated to RT2 in the interference regime. When the manipulation was applied to the task performed second (Figure 4E), the first task was unaffected but the second task showed an additive effect not absorbed at short SOA values. This effect is what would be expected from bottleneck manipulations. The statistical significance of these observations was evaluated with a series of ANOVAs using the R software package (http://www.r-project.org/) (Table S1).

The response times histogram for SOA = 0 ms is displayed in Figure 5 (A, B). The results of the model capture an important experimental observation that the variability in RT2 is higher at short SOA, as RT2 accumulates the variability of both tasks. Response times for T2 become faster and less variable as SOA increases, as seen by plotting the cumulative response time distributions for varying SOA (Figure 5F) [18]. Interference and seriality are also observed in the scatter plots of RT1 vs. RT2, for different SOA values: for short SOA values RT2 is tightly correlated to RT1 indicating that RT2 is sequentially locked to Task 1 completion. For long SOA values, RT1 and RT2 become independent measures (Figure 5 C–E).

### Effects of Noise and Oscillatory Inputs on Response Times

The previous results showed that our model can explain the precise shape of response time distributions in dual-task performance. Here we investigate the underlying physiological markers which result in such distributions, i.e. the relation between neuronal and response time variability. All neurons in the model receive strong background Poisson inputs, which assures a spontaneous activity of 2–5 spikes/s. We hypothesized that in trials in which input noise in the sensory neurons coincides with stimulus presentation (presented for 100 ms) response times would be faster. We also hypothesized that in the case of low-frequency noise (∼5Hz), the coincidence effect of external-stimulus and internal noise fluctuations, should manifest in a phase-locking relation of stimulus presentation to internal rhythms, as observed in both psychophysical [63], [64] and neurophysiological [65] experiments.

**Figure 6:**
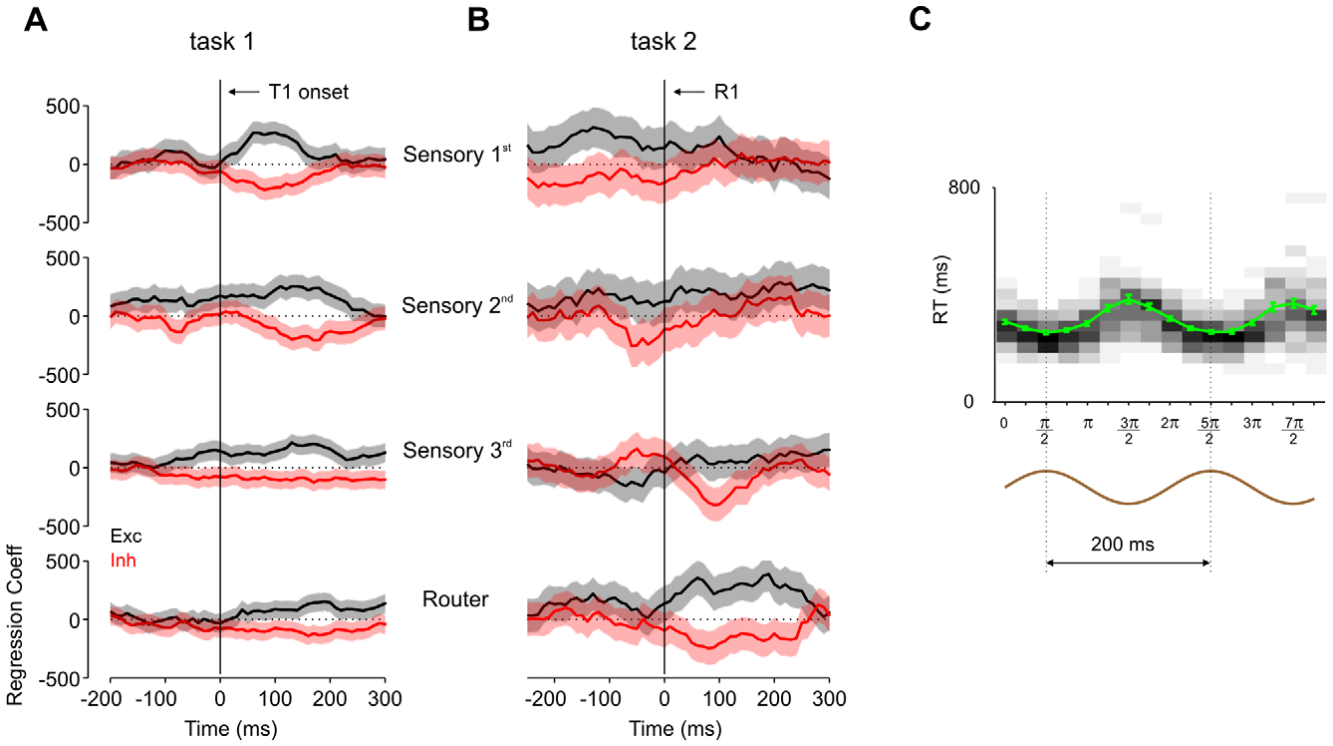
Response time sensitivity to stochastic fluctuations and low-frequency oscillations. (A,B) Coefficients of the linear regression model used to relate fluctuations in background inputs to response time variability. Black traces correspond to stimulus-selective excitatory populations at different processing levels, as indicated in the figure's legend. Red traces correspond to inhibitory neurons within the same area. Shades depict 95% confidence intervals. A positive coefficient means that higher activity due to noise leads to faster responses. (A) Estimates for Task-1 sensory and router populations, with RT1 as the independent variable. The x-axis indicates the time relative to stimulus onset, and thus positive values correspond to noise fluctuations occurring after stimulus onset. (B) Estimates for Task-2 sensory and router populations, with RT2 as the independent variable. Here, neural activity across different trials was locked to response 1 before the regression analysis. (C) Mean response times (green trace) for single-task simulations as a function of the phase between stimulus onset and background noise. The x-axis depicts the phase of stimulus onset relative to the background fluctuation (brown trace, bottom), and the y-axis depicts the mean response time in milliseconds. Error bars indicate the standard error of the mean. 50 trials were simulated for each individual phase. Also shown in grey-scale are the response time histograms (bin size of 40 ms).

We first used a general linear regression model to investigate how noise fluctuations affected response times in the PRP. The explanatory (independent) variables were external noise fluctuations for each population group and temporal bin, and the response (dependent) variable was either RT1 (Figure 6A) or RT2 (Figure 6B).

We simulated 900 trials of the PRP for an SOA of 50 ms. For each trial, the population average of 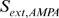 - dynamic gating variable mediating background AMPA currents (see Materials and Methods section) - was measured every 1 ms, assigning a value of 0 if its value exceeded the median value over all trials, and a value of 1 otherwise, independently for each population and time step. Independent variables were obtained by averaging these values within windows of 100 ms. Similar populations - for example, all neurons in the first level of the sensory hierarchy selective to the same stimulus - were averaged together. A positive regression coefficient means that higher activity of a group of neurons leads to faster responses.

The time-course of the coefficients of the regression (Figure 6) showed a very clear temporal organization. For Task-1 sensory neurons (Figure 6A), fluctuations in the first sensory level which were coincident with stimulus presentations were highly predictive of RT1. On the contrary, fluctuations beyond this window were essentially independent of response time. In successive stages of the hierarchy the window of correlation was delayed.

As we showed previously, RT2 variability accumulates RT1 variability (due to changes in the onset of the routing of T2) and intrinsic variability of the T2 routing process. To understand the impact of noise on each of these processes, we measured the time-course of the noise input to Task-2 responding neurons locked to the response to Task 1 (Figure 6B). Significant noise contributions were observed before the integration onset (Figure 6B, upper panel), suggesting that although sensory integration is delayed during the PRP, fluctuations in the memory trace of S2 during T2 queuing or before have an influence on RT2.

Thus, spontaneous Poisson-noise fluctuations were effective when they coincided in time with external stimulus currents. If noise currents were carried by low-frequency oscillations [66] this effect could result in phase locking of RTs to the rhythmic oscillatory activity. We tested explicitly this possibility by running single-task simulations where excitatory neurons in the first sensory level received a low-frequency (5 Hz), low-amplitude (0.06% of the external background noise), oscillatory input. This additional input resulted in a small synchronous fluctuation on top of the large external background input. The phase of the stimulus onset relative to the background rhythm was varied across trials in order to study its effects on average response times and their distributions (Figure 6C). The relative phase between stimulus onset and rhythmic background activity had a marked effect on response times, compatible with recent experimental findings [65] and theoretical proposals [66] linking low-frequency oscillations to attentional selection. Our model provides a simple physiological explanation of why phase-locking stimulus to low-frequency oscillations may result in shorter response times. When the phase is such that the peak of noise fluctuations coincides with stimulus presentation, the stimulus is enhanced and this reduces response time. On the contrary, when stimulus presentation coincides with the valley of noise oscillations, input to the router is less effective and response times are longer.

### From the PRP to the Attentional Blink

Behavioral experiments which have combined the basic features of different manifestations of central processing such as the PRP (two rapid responses) or the attentional blink (extinction of a second rapidly presented stimulus) have suggested that both forms of processing limitations may arise in part from a common bottleneck [67]–[70]. The main differences between the PRP and the AB is that in the PRP a speeded response is required to the first target and, most importantly, that in the AB the visibility of the second target is reduced, generally by masking it or by embedding it in a rapid visual serial presentation (RSVP).

To evaluate whether our model could, without modification, also account for AB experiments, we studied the effect of a mask applied after T2. The mask was modeled as a brief stimulation of non-specific excitatory cells in the first layer of the sensory hierarchy, thus modeling the activation of a neural representation competing with the target T2 [71]. The mask lasted 100 ms and was presented immediately following T2. In the majority of AB experiments, both T2 and the T1 are masked. Here, for direct comparison with the PRP simulations, we considered a special AB case in which the T1's fleetingness is obtained by virtue of its weak strength, rather than masking [72].

**Figure 7:**
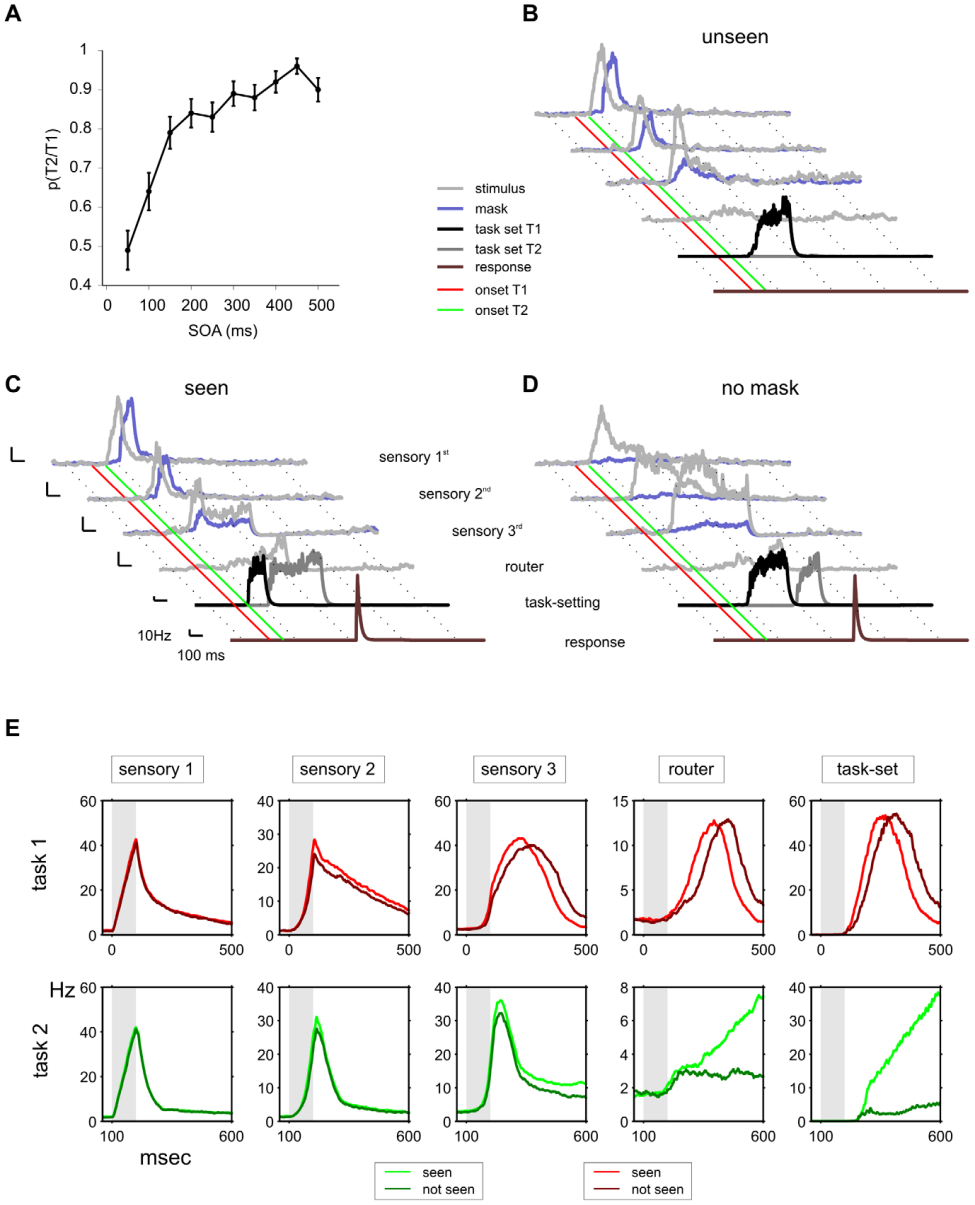
From the PRP to the attentional blink: masking effects on visibility. When T2 is masked the model displays characteristic aspects of AB experiments. (A) Probability of responding correctly to T2 given T1 correct, for varying SOA (error bars depict the standard error of the mean). (B–D) Single-trial population firing rates of relevant populations for trials with *seen* and *unseen* T2. The average response of neurons selective to T2 (grey traces) and to the mask (purple traces) is shown at different sensory levels. The average activity of task-setting excitatory neurons selective to T1 is plotted in black, and motor neurons for the correct response to T2 are plotted in brown. The red line indicates the onset of T1, and the green line the onset of T2. (B) An *unseen* trial (the network fails to respond to T2) with SOA = 100 ms. The mask interferes with the reverberation process of T2 and activity in the last sensory areas decays before it can be recovered by the activation of T2 task-setting network. (C) A *seen* trial (the network responds) with the same SOA as in panel A (100 ms). The task-setting network disengages faster from T1, accelerating T2 task setting activation which permits integration before T2 sensory memory has faded out. (D) A trial with SOA = 100 ms and no mask (a PRP trial). In the absence of a backwards mask, the traces of T2 in sensory areas last longer and thus routing of T2 occurs despite the delayed engagement of task-setting neurons. Population firing rates were calculated by convolving the spike raster with an exponential causal kernel of 20 ms. (E) Average neural activations of selected populations for 129 *seen* and 64 *not seen* trials, for SOA = 100 ms. Each column indicates the averaged activity of one selective excitatory population, as indicated in the top of the panel, for the first (top row, red) and second (bottom row, green) tasks. The two traces shown in each cell show the activity of the same population when a correct (light green and red) or absent (dark green and red) response is given to T2. Averages were calculated by filtering the instantaneous population firing rate with an exponential causal kernel with a time constant of 20 ms and averaging across trials.

We simulated 100 trials at each SOA value, varying the SOA between 50 and 500 ms at 50 ms intervals. In contrast to the previous PRP simulations, when the SOA between T1 and T2 was short we observed a small (but significant) number of errors and, most importantly, a large number of trials in which the network failed to respond to T2 (Figure 7A). For simplicity and to follow the convention of prior experimental work, we refer to trials in which the network responds correctly as *seen*, and those in which it fails to respond as *unseen*. For example, at SOA = 50 ms we obtained 49±5% *seen* trials, 47±4.99% *unseen* trials, and 4±1.96% errors; for SOA = 500 ms, we obtained 90±3% *seen* trials, 9±2.86% *unseen* trials, and 1±0.01% errors. As observed in the Attentional Blink and in mixed AB-PRP paradigms, the brief mask after T2 is only effective when T2 is presented within a short temporal window – typically of around 500 ms – following T1 presentation.

For short SOA values, the network exhibits a highly stochastic behavior: the same configuration of stimuli and SOA may lead to *seen* or *unseen* responses depending on the inner state of the network. Figure 7B–D shows the time-course of activity of a representative *seen* and *unseen* trial and reveals the cause of the blink. In the *unseen* trial, RT1 was longer and thus at the moment in which inhibition of T2 task-setting neurons was released, T2 sensory activation had faded out. As a consequence, T2 task-setting neurons failed to respond and this impeded the integration and routing of T2. This can also be seen when averaging across all trials (for an SOA of 100 ms) according to whether the network responded or failed to respond to T2 (Figure 7E). T2 non-responded trials resulted – on average - from a delayed response of the T1 task setting neurons. This observation establishes a concrete prediction for the dynamics of routing neurons in a AB experiment and is consistent with physiological and behavioral experiments which have shown that the extent of T1 processing has an impact on T2 visibility [69], [73], in accordance with the behavior of the sequential bottleneck model.

The interpretation of our results is that the mask results in an accelerated exponential fading of the representation of T2 stimulus in short-term memory [74], [75]. As a result, if the waiting time of T2 is too long, due to the concurrent processing of T1, the remaining activation is insufficient to ignite the router and task-setting neurons and the network fails to respond to T2. Consistent with this interpretation, we verified that early responses evoked by the second stimulus in *seen* trials showed a small, but significant effect in the amplitude – but not in the latency - of the transient responses when compared to *unseen* trials (Figure 7E). These small fluctuations are strongly amplified in the router and task-setting neurons, which show an almost all-or-none difference (Figure 7E). This result is consistent with electrophysiological experiments of the blink and the PRP which have observed a modest effect in early sensory components and a massive all-or-none effect in late P3 components [73], [76], [77].

A series of experimental observations have shown that the AB is attenuated (i.e. the probability of seeing T2 increases) with increased T1 strength. For example, the blink is attenuated when a blank is placed after T1, i.e. masking is delayed [10]. This observation is in contradiction with pure T1–T2 competition models of the AB since these models predict the opposite effect: increased T1 strength should result in a reduced likelihood of perceiving T2 [78], [79]. However, it seems compatible with our network operation, since a stronger T1 stimulus should result in a faster conclusion of Task 1, increasing the probability of retrieving the second stimulus before it has fade out.

We examined this hypothesis performing two different simulations. First, we increased the strength of T1 by 10% relative to the previous PRP and AB simulations. This resulted in an attenuated AB for the second task (76±4% correct vs. 49±5% correct without the manipulation; p-value <0.0005; 100 trials at a fixed SOA of 50 ms). Despite perfect performance for T1 in these simulations, RT1 was smaller when T1 was stronger (with strong T1: RT1 = 318±5 ms; without the manipulation: RT1 = 396±9 ms; p-value<0.0005). Thus increasing T1 strength decreases RT1 and increases the probability of retrieving the second stimulus.

The second manipulation, conversely, involved masking the first target T1, simulating the most typical AB paradigm in which both T1 and T2 are masked. As for the first manipulation, 100 trials were simulated at a fixed SOA of 50 ms and we now added a mask identical to the one previously used for T2. In this condition, performance in the first task was still accurate (92±3% correct) while T2 visibility was decreased significantly (26±4% correct). This effect can be understood by the increased latency of the inhibitory signal following routing of T1, which increased RT1 from 396±9 ms in the unmasked condition to 869±50 ms when T1 was masked.

In summary, our simulations show that T1 manipulations that facilitate the first task and therefore reduce its duration have the effect of reducing the attentional blink for T2, as experimentally observed [5], [80]. Since RT1 is typically not measured in most AB tasks, where the task is to covertly commit T1 to memory for delayed report, only the reduced blink for T2 would have been noticed experimentally – but our network suggests that, if RT1 was measured by an on-line task, then the reduced AB would be replication and would be mediated by a faster RT1.

## Discussion

### Summary of Results

The present model constitutes, to our knowledge, the first spiking-neuron model of a global architecture capable of simulating the entire sensory-motor chain of processing in a dual-task setting. We could explain the detailed dynamics of behavior (including both mean RTs and RT distributions) during dual-task-performance, by simulating a large-scale network of realistic neurons, comprising about 20.000 spiking neurons and 46.000.000 synaptic connections. For consistency with the majority of previous PRP experiments, we simulated an experimental design in which stimuli involve distinct sensory modalities and the responses distinct effectors. Under these circumstances, interference occurs exclusively at the routing stage, commonly referred to in psychology as the response selection stage [4]. The central aspect of our model is a detailed neuronal implementation of this flexible “routing” and how it manages to change from one task to another in hundreds of milliseconds, using an area that maps stimuli onto responses which we have termed the router network. The model capitalizes on a number of existing elements: (1) perceptual attractor networks capable of encoding stimuli and maintaining them in an exponentially decaying buffer [62], [71], (2) an accumulation-to-threshold mechanism, comprising both recurrent neuronal assemblies [36] and a thresholding device inspired by the architecture of basal ganglia [81]; (3) a control network comprising rule-coding units capable of modulating other areas in a top-down manner [32], [45], [55], [82]–[85]; (4) the concept of a routing circuit implemented by neurons with broad connectivity, capable of transiently interconnecting other brain processors in a flexible manner [33], [47], [86]–[89]. The novel aspect of the present simulations is to integrate these theoretical constructs into a global functional architecture. We observed that the interplay between these control and routing mechanisms resulted in a central limitation during dual-task processing, which manifested itself either as a delay in the second task (PRP), or a complete interruption of the processing of a second target (Attentional Blink).

Based solely on the known dynamics of neurotransmitter receptors, the model reproduces, in a quantitative manner, a large number of behavioral observations of dual-task interference (see [17], [18], [35]):

1. A sequential delay in RT2. This delay decreases with a slope of −1 as SOA increases reflecting a sequential bottleneck.
2. The absence of any effect of the second task on response times to the first task (mean and distribution).
3. Strong correlations between RT1 and RT2 which progressively diminish as SOA increases.
4. Distinct interference patterns associated with different task manipulations: changes which affect the sensory delay processing of Task 2 are absorbed during the slack time separating task 1 and task 2, while changes which affect the accumulation time (i.e. central processing in the router) propagate additively.
5. Switch from the PRP (delayed response to T2) to the blink (an absence of the response to T2) by adding a mask after the T2 stimulus.
6. An increase in blink probability when T1 visibility is reduced.

These results are in full accordance with the central interference model [17], [35], [90], by which certain processes are carried out in parallel and routing and accumulation are intrinsically serial. Our model provides a detailed neuronal implementation of this classical psychological model and makes many new predictions for the neurophysiological correlates of the PRP.

### Comparison with Previous Neuroimaging Studies

Several brain-imaging experiments implicated a number of cortical systems in the PRP phenomenon. The cerebral basis of processing bottlenecks has been investigated with Event Related Potential studies (ERPs), which have shown that the PRP results in reduced and/or delayed components [91]–[97]. Using time-resolved fMRI [98]–[100], Dux and collaborators showed a slight delay in the peak fMRI activity in prefrontal cortex during a PRP paradigm [101], implying that the PFC was one of the fundamental nodes responsible for the central bottleneck of information processing. Recently, using both time-resolved fMRI and high density ERP recordings we could fully parse the execution of two concurrent tasks in a discrete sequence of processing stages. The ERP analysis demonstrated that a late P3-like complex is in fact delayed by an amount comparable to the PRP effect on RTs, and time-resolved fMRI confirmed that the PRP delayed parietal and prefrontal activation by several hundreds of milliseconds [77]. The notion that the global P3 indexes a late capacity-limited central stage fits with results from the AB. As we could show in the simulations the main difference between the PRP and the AB can be accounted for solely by the masks used to produce the AB, which interfere with the local memory of T2. The result is that T2 processing is not merely delayed (PRP), but erased and it therefore escapes from consciousness. During AB, the initial ERP components up to about 270 ms are essentially intact, but the P3 component is essentially abolished [73], [76], [102], [103]. The P3 component can only be detected in seen trials, in an all-or-none fashion [73], [104]. We observed this precise dependence for the activity of routing neurons and the onset of task-setting neurons, suggesting that the P3 is likely to constitute a large-scale electrophysiological marker of the router system. Also, as indicated by our simulations, increased latencies in T1 processing resulted in higher probability of the second target being blinked [73], [105], [106]. Direct comparison of AB and PRP paradigms suggests that both affect the same P3 component [95].

The spatial resolution of EEG is very imprecise and thus a better characterization of the locus of central processing bottlenecks in the brain comes from fMRI studies, which have pinpointed a broad parietofrontal network that exhibits various manifestations of central capacity limits [67], [107], including the AB [67], [105], [108] and the PRP [77], [101], [109], [110]. This network is ubiquitously activated by a large variety of goal directed tasks [107] suggesting that it plays an important role in flexible routing information between remote neuronal representations.

Our network postulates a hierarchical organization of this system: neurons controlling the whole-task structure (order network) gate neurons controlling the individual tasks (task-setting network), which, in turn, gate the routing from the sensory representations to the motor intention stage. Such a hierarchical organization has been demonstrated in humans in the prefrontal cortex as the Broca region and its homologue in the right hemisphere implement executive processes that control start and end states as well as the nesting of task segments that combine in hierarchically organized action plans [52], [111]–[114]. A hierarchical organization involved in planning of complex sequential tasks has also been found in non-human primates [113], [115].

### Emergence of Seriality in Cortical Networks which Perform Flexible-Task Settings and Scaling of the Model

Understanding the emergence of serial behavior in the human brain is an important and central theoretical question in cognitive psychology as modularity and parallel processing are hallmarks of brain computations. Different authors have proposed cognitive architectures that can explain how components of the mind work to produce coherent cognition [14], [24], [86], [116]–[118]. Concrete implementations of these ideas have shown that these coherent states which transiently bind together existing modular processors naturally result in serial behavior [14], [43].

Here we have tentatively proposed that seriality in dual (or multiple) task performance results from the necessity to establish a task set through the activation of a “router” network. This router network is shared by all sensory-motor mappings and its activity can, potentially, code for a virtually infinite number of possible tasks. A task-setting program acts as a gate, permitting routing neurons to propagate information if they receive the appropriate sensory input. This system acts as a control mechanism that avoids erroneous, conflicting or unwanted stimulus-response associations. We showed that a concrete implementation of such a control system results in serial behavior of the routing process when probed in dual-task situations.

In our network, seriality and its behavioral manifestations, the PRP and the Attentional Blink, emerged from competition between task-setting neurons which, through a lateral inhibition process, prevented the simultaneous activation of two task settings. This form of control is necessary to ensure correct task performance in conflicting mappings - as classically demonstrated in the Stroop paradigm in which the same stimulus may lead to distinct responses according to task requirements [119]. While this mechanism is strictly required only in conflicting response mapping situations, which is not the case in our present simulations, it is possible that it has emerged as a ubiquitous mechanism in control networks to assure correct task performance. Seriality in non-conflicting tasks would therefore emerge as a consequence of the need for a flexible mechanism linking stimuli with multiple responses according to context [28], [29].

Another possible origin of seriality relates to the coding properties of the router (for a simple illustration see Figure S6). Here we have explored a comparatively simplified situation of a small number of tasks, stimuli and responses in which all possible routings were coded by distinct neural populations. This mechanism would result in a combinatorial explosion in a more realistic setup, arguing that the code of router neurons should be distributed, i.e. each routing scheme should be encoded in a large population of neurons. This is consistent with many findings in prefrontal cortex neurons which have found that a large fraction of neurons respond to virtually all tasks [83]. In this scheme, the precise pattern of active and inactive neurons determines the code and thus superposing two routing configurations (of two distinct tasks) should result in a mixture leading to erroneous mapping properties. Avoidance of incorrect mappings in a combinatorial router can be implemented by the same mechanism shown here, leading to serial routing in the composition of flexible task settings (Figure S6).

### Comparison with Alternative Implementations and Existing Models

Previous modeling efforts have established cognitive architectures which can account for human complex problem solving [14], [24], [116]. The adaptive control of thought–rational (ACT-R), for example, proposes a theory of distinct modules that interact with each other to produce coherent cognition [14]. While ACT-R is based on a sequential scheme, the temporal constant of the sequential step in ACT-R and in the PRP are not comparable: in ACT-R, productions (if-then structures representing procedural knowledge) fire approximately every 50 ms, about five times faster than the PRP delay. The 50 ms delay of individual productions is consistent with other experimental approaches which have suggested a discrete organization of cognition at a frequency close to 13 Hz [120]. These observations of ∼50 ms productions and the comparably slower ∼300 ms PRP delay can be reconciled by modeling the entire routing program as a sequence of productions, as in the ACT-R implementation of the PRP of Byrne and Anderson [25]. Sensory modules in the ACT-R involve a two-layer structure, a visual module (mapped to occipital/temporal regions) and a visual buffer (mapped to parietal regions). The visual buffer incorporates a selection mechanism that determines the contents of the visual system which will be available to other processors. Our model provides a concrete neuronal implementation of these mechanisms. In our model, the sensory hierarchy acts as a module which can select and maintain information locally (unless a subsequent element such as the mask overrides the buffer). This information can be broadcasted to the rest of the network. Similarly, in ACT-R the selection of actions is achieved by a loop that mimics the Basal-Ganglia- cortical connections. By building up on previous architecture for thresholding and gating sensory information through striatal-cortical interactions [44] our model provides a neuronal implementation of these mechanisms.

The router circuit in our model builds on previous computational models which have studied the role of contextual signals on transient sensory-motor mappings [30], [33], [121], [122]. Salinas (2004) showed that a linear read-out of sensory input could result in arbitrary sensory-response mappings if sensory responses are modulated by (a non-linear) contextual influence. A concrete implementation of flexible mapping by rule-setting contextual signals was developed by Deco and Rolls [47], [123].

In the present model, the router binds sensory and motor representations. Similar conceptions of flexible routing circuits have been applied to other instances of information binding such as, linking the attributes of an object in pattern recognition [89] or linking discrete objects to temporal contexts through distributed representations as recently proposed by Wyble and Bowman [124]. Olshausen and colleagues implemented a routing scheme in a set of control neurons which rapidly modify the strength of intra-cortical connections to implement the attentional gating of information flow from early visual representations to a higher level object-centered reference frame [89], [125]. The SAIM model of selective attention [88], [126] has shown how this ‘dynamic routing’ model can be extended to account for a wide range of results of visual experiments with competing stimuli in space, i.e. neglect [127] or in time, i.e. inhibition of return [88] in both normal and impaired subjects. The SAIM model [88] shares many features with our network: it implements a routing neuron which is modulated by a control (task-setting) network and thus acts as a coincidence-detector of a task-setting program and current sensory state. Recently, Heinke and collaborators showed how the SAIM model can be implemented with spiking units [126].

Our network provides an implementation of simple boxological models of dual-task execution in the PRP [17], [34], [35]. While very simple, these models have established a vast range of predictions in behavioral experiments regarding the precise functional dependence of RTs with SOA and how these functions should change with different manipulations. By incorporating ideas of models of decision making, we previously generated a schematic model that accounts for the entire distribution of RTs and how it changes in the interference regime [18]. Here we have shown that these ideas can be implemented robustly in realistic network architecture.

A critical aspect of our network is that while the router is occupied by T1, the T2 stimulus was maintained in the recurrent activity of high-level sensory units, thus forming a memory which remains local because it cannot activate the router. This coexistence of parallel mechanisms – a cascade of sensory processes which encode the stimulus - and of serial bottlenecks – queuing by the routing process - constitutes a hallmark of PRP observations. Our network implemented this local memory as a local attractor showing progressive integration and exhibiting a metastable form of memory that could be maintained for a few hundred milliseconds. According to this proposed mechanism, the memory trace remains stored in a local network and is relatively fragile as it can readily be overridden by a mask. The critical observation is that the mask can only override processing of T2 if it the router is occupied by T1.

To our knowledge, our model is the first one to propose a concrete neural implementation of the mechanisms leading to the PRP. In contrast, several computational models have been recently proposed for the attentional blink [43], [78], [128]–[130]. Two current explanations include the simultaneous type serial token (ST2) model [78] which proposes that access of sensory representations to working memory is gated by an episodic-driven attentional signal and the *boost and bounce* model [130] which suggests that a target initiates an attentional boost which is interrupted when the trailing task-irrelevant stimulus is accidentally boosted. Our model shares with the ST2 model the idea of gating of a router-system and with the boost and bounce model that task-setting activation is not a phasic event, but rather, can stay active until it is inhibited by a termination signal.

We emphasize that our model does not intend to give a detailed account of all the findings from attentional blink experiments, but instead to show how the same mechanisms that lead to delayed responses in the PRP can lead to missed targets in the AB. Recent reviews of the extensive AB literature argue for a multifactor origin in this processing deficit [131], and thus it might be impossible to pinpoint a single mechanism behind the full diversity of experimental findings (although see [132], [133]). Nevertheless, our results show that limited capacity operations – as the one implemented by our router/task-setting network – may play a central role in the attentional blink [72], [134].

One aspect of the attentional blink phenomena which our model fails to replicate is the relative increase in performance observed at very short SOA (∼100 ms), an effect known as lag-1 sparing [5]. This effect is not observed when T1 and T2 involve different modalities [135] (as in our simulations of the AB) or spatial locations [136]. Recent experiments show that the sparing can even be spread to several targets presented rapidly without intervening distractors [137], [138], suggesting that the unit of selection of a serial attentional process is not the individual target but an extended event which may include several rapidly presented targets [132], [139], [140]. This grouping does not happen without a cost, since order swapping and performance tradeoffs between different targets do occur [78], [141]. In our model, the task-setting configuration is sustained until information is routed to the motor system, and thus it might be possible to extend the present model such that more than one target in a RVSP benefits from the same task-setting configuration. Processing a temporally extended event encompassing several targets would require broadening – in feature space - the action of the task-setting network as well as making the router/task-setting complex capable of flexibly routing information not only to motor areas but also to mnemonic [142] or sensory areas in order to achieve recursive computations.

In fact, we see the extension of the present model along the lines just discussed: the different types of neurons used in our implementation (briefly reviewed in the next section) have been found in the awake behaving monkey and may serve as a basis from which to construct complex cognitive programs, as those implemented in systems like ACT-R [3] or SOAR [143] - but with a stronger grounding on neurophysiological findings [144]. In this implementation, we see router neurons as capable of accumulating evidence not only towards a motor response, but implementing a full production system [145], [146] where stochastic rules are selected according to the information contained in different mnemonic systems which are in turn updated by external stimuli and by the action of the productions themselves. These ideas will form the basis for a future extension of the present model to flexible series of chained tasks.

### Comparison to Previous Electrophysiological Studies and Novel Predictions

Most, if not all, types of neurons used in our implementation have been observed in studies that measured single-neuron activity in awake behaving monkeys during single-task performance. Here we will briefly mention the main types of neurons in the various areas of our model and compare them to neurophysiological data, a comparison that will have to remain somewhat superficial as we cannot attempt to discuss the precise relationships between the variety of tasks employed in the neurophysiological studies and the PRP task implemented here. Firstly, the properties of the sensory areas of our model are consistent with what is known about representations in areas of sensory cortex. Neuronal activity in low level sensory cortex is largely (but not entirely) determined by the incoming sensory information [147], while neurons in higher areas carry information about the behavioral relevance of stimuli, as well as traces of stimuli to be remembered [148]. Secondly, neurons in areas of parietal and frontal cortex have response properties consistent with the routing process proposed by our model. Many of these cells are tuned to categories of stimuli that are associated with a particular behavioral response [149]–[151] and integrate evidence in favor of one of a number of possible actions until a threshold is reached, just as is required by the model's router [152]–[154]. Thirdly, some neurons in the frontal cortex only respond if a particular stimulus maps onto a particular motor response, but not when the same stimulus or response is part of a different stimulus-response mapping [60], and yet other prefrontal neurons code abstract rules [84]. Clearly, the response properties of these neurons are in accordance with the model's task-switching network. Finally, neurons in the motor response selection stage of our model have either a gradually increasing activity before the response or they respond with a sharp burst at the time of the response. Neurons with gradually increasing activity before the motor response and cells with a motor burst are indeed observed in areas of the motor cortex [155], [156] as well as in the basal ganglia [157]. These results, taken together, indicate that the types of units required by our implementation are broadly consistent with the types of neurons that are observed in neurophysiological experiments.

Our network can also explain timing and latencies of the sequence of events identified in single-task physiological experiments in monkeys [158]–[160] and humans [161]. Accumulation of information about the upcoming response influences the firing rate of routing neurons at a latency of about 200 ms, a latency that may be relatively fixed for a given task [162]. This latency cannot be explained solely by synaptic delays, since measurements of conduction velocity of cortical feedforward and feedback connections showed that they can be rapid, even faster than intrinsic connections within a cortical area [163], [164]. A previous neurophysiological study showed that the onset of response modulation in the visual cortex depends of the sequencing of subtasks, with later modulation for subtasks that occur later in a sequence [165]. Our model grasps this observation: the latency of the response of routing neurons depends on the order in which the two subtasks are executed (Figure 3B–C). The present results suggest that the latency of feedback modulation may reflect the time required by the network to settle into a brain-scale state of coherent activity [18], [87], which in our model is reflected by a coherent pattern of activity across sensory, router, and task-setting networks coding different aspects of the same subtask.

Our observations also raise a note of caution on the interpretation of processing latencies from physiological data. A concrete example is conveyed in our model by the measurement of activity in the routing neurons. Spiking activity shows a clear sequential scheme: routing neurons of T2 start integrating only once routing of T1 has completed (Figure 3B). Thus, the latency at which spiking activity exceeds a certain threshold constitutes a physiological marker of the PRP effect. The picture is quite distinct if one would measure synaptic router activity (Figure S3). During the time in which T1 is being routed and T2 is being buffered, T2 sensory neurons spike and project silently (i.e. without evoking spiking responses) to router neurons. Hence synaptic activity in T2 router neurons increases during T2 compared to baseline. A consequence of this observation, which may be of relevance beyond the specifics of this study, is that timing analysis based on synaptic or spiking activity yield qualitatively different observations. Various studies have simultaneously measured different markers of neurophysiological activity such as multi-unit activity (MUA), laminar current-source density (CSD) and local field potentials (LFP) [166] and fMRI [167] or EEG [168]. Multimodal interactions have been shown to display such a mixed effect in response latencies. Primary auditory cortex shows a clear CSD response to somatosensory stimulation, without observable changes in the spiking response as measured by MUA [169]. Computational models may be a useful link to bridge information gathered at different scales.

Our data showed that fluctuation in response time could be accounted by the dynamics of noise fluctuations in relation to the timing of stimulus routing (Figure 6). When noise is oscillatory, this is determined by a precise phase relation. Our model does not explain how this relation can be entrained. Neurophysiological data of multi-sensory integration suggests that somatosensory stimuli can reset the phase of ongoing oscillations in primary auditory cortex such that auditory stimuli are boosted if presented during the high excitability phase [169], [170]. Also, it has been shown that neuronal oscillations can entrain to environmental rhythms improving discriminative performance and decreasing response times [65], [66]. As mentioned, these aspects lie outside the scope of the present model.

The correlates of the bottleneck have yet to be studied at the single cell level and our simulations therefore generated a number of new predictions that could be tested in future neurophysiological experiments. First the model establishes the existence of routing and task-setting neurons with well distinct dynamics and connectivity with different neuronal populations. At the anatomical level, routing neurons should receive inputs from all sensory modalities and from task setting neurons. At the functional level, they should be characterized by their firing in response to specific conjunctions of stimuli and responses, a preference which may change dynamically according to task context, on a time scale of about 100 ms or more (for supporting evidence, see [60], [113]). Task-setting neurons should engage in a competition such that two task-setting programs or routing schemes cannot coexist in time. This should avoid unwanted mappings but also causes an inertia which results in relatively slow switching (>100 ms) from one task-setting to another leading to seriality in the routing process. In a PRP experiment, neurons coding for the memory T2 stimulus should show a characteristic temporal profile, comprising (1) a phasic sensory response, time-locked to actual stimulus presentation, (2) a sustained response exhibiting a slow exponential decay, and (3) a late amplification at the time when task 1 routing is completed and the router neurons of task 2 become active. On the contrary, the onset of router and task-setting neurons of Task 2 should be delayed at short SOA, with a delay that should decrease with SOA because task 2 router neurons are released from the inhibition of task 1 as soon as it is completed. In trial-by-trial comparisons, at short SOA values, the onset of router and task-setting neurons of T2 should be locked to the response time of the first task. While sharing the onset, the model predicts distinguishable time-courses of activations for router and task-setting neurons. Task-setting neurons should show sustained high-levels of activation throughout the duration of the task while router-neurons activity should ramp to a critical threshold. In an AB experiment task-setting neurons of T2 should be active both in *seen* and *unseen* trials. Only in unseen trials should the memory of T2 fade below a threshold (either due to fluctuations in transient response or in the durations of the memory due to the extension of T1) impeding routing and broadcasting to the rest of the network. These predictions will become testable once an awake animal model of dual-task performance is defined.

## Materials and Methods

### Neuron Model

The model contains 21,000 neurons and 46,634,400 synapses. Neurons were either excitatory or inhibitory. All neurons were modeled as conductance-based leaky integrate and fire units. The membrane potential of each cell below the threshold for spike generation is described by:
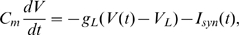
(1)where 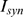 is the total synaptic current flowing into the cell, 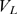 = −70 mV is the resting potential, 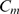 is the membrane capacitance (0.5 nF for pyramidal cells and 0.2 nF for interneurons), and 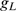 is the membrane leak conductance (25 nS for pyramidal cells and 20 nS for interneurons). The threshold for spike generation was set to −50 mV. The reset potential after spike generation is −55 mV, and the refractory period is 2 ms for pyramidal cells and 1 ms for interneurons.

All neurons receive large amounts of background synaptic activity which determines the level of spontaneous activity. External inputs and background activity are mediated exclusively by AMPA receptors.

Recurrent excitation is mediated by AMPA and NMDA receptors, and inhibition is mediated by GABA receptors. The total synaptic currents are given by:
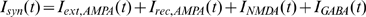
(2)in which
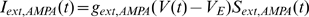
(3)

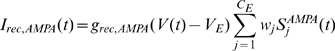
(4)

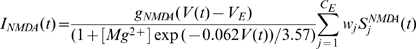
(5)

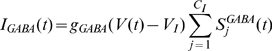
(6)where 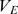 = 0 mV and 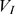 = −70 mV. The extracellular magnesium concentration 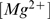 = 1 mM controls the voltage dependence of NMDA currents [171]. 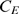 and 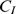 are the number of excitatory and inhibitory inputs, respectively. The values of the synaptic efficacies *g* are given below. The dimensionless factor *w* controls the strength of recurrent connections between neurons with similar response properties (see below). 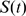 in equations 3–6 is the gating variable - or fraction of open channels –updated according to the activity of the presynaptic neuron *j* and the identity of the receptor mediating the transmission. The dynamics of the gating variables are as follows. When a neuron receives a presynaptic action potential the appropriate gating variable *s* is increased. Otherwise, these variables decay exponentially. For AMPA and GABA receptors:
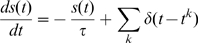
(7)


For NMDA receptors:
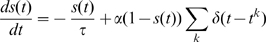
(8)where 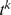 is the time of presynaptic spike *k* and α = 0.63 controls the saturation properties of NMDA channels. The decay time constants are τ*_NMDA_* = 100 ms, τ_AMPA_ = 2 ms, and τ_GABA_ = 10 ms.

### Neural Architecture

Neurons are grouped into homogeneous populations. A total of 84 unique populations were included in the simulations. In sensory and routing areas these homogeneous populations were grouped into larger groups, forming local *modules* as used in previous studies [36], [37].

#### Sensory and routing networks

Sensory areas are modeled through a hierarchy of modules, to account for convergence and increased receptive fields at higher levels of processing [53], [172]. Stimuli from the two tasks in the PRP task excite different sub-sets of selective neurons. Thus, in each module we included two selective population (80 neurons each), one group of inhibitory interneurons (200 neurons), and one large non-selective population (640 neurons) grouping all stimuli not relevant to the task. Synaptic efficacies in local sensory and routing networks are the same as in [36] (in nS): for pyramidal cells, 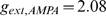, 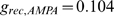, 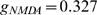, 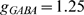; for inhibitory cells, 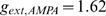, 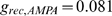, 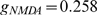, 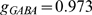. All synaptic connections between connected neural populations and within the same population are all-to-all.

Within local modules, connections are structured according to a “Hebbian” learning rule: coupling strength between pairs of neurons is considered to be high for neurons inside a selective population, and low when connecting neurons from competing populations. Specifically, for synapses connecting neurons within the same selective population, a potentiated weight 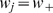 was adopted, where 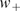 is a number larger than one. For connections between distinct selective populations, and from non-selective to selective populations, 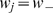, where 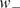 is a number smaller than one. In order to maintain the spontaneous activity of the network as 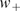 is varied [173], 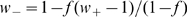, where 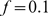 is the fraction of excitatory selective cells. For all other connections 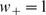. In the sensory hierarchy 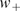 increases at higher levels in the cortical hierarchy, with values [1.8, 1.81, 1.94] for levels 1 to 3 respectively.

Feedforward and feedback connections have different degrees of specificity. Feedforward connections are highly specific: neurons from one excitatory population project exclusively to one excitatory population in the immediate higher level. These connections are mediated exclusively through AMPA receptors (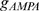 = 0.11 nS for selective populations and 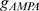 = 0.0138 nS for non-selective populations; different efficacies were adopted to compensate for the different number of neurons in selective and non-selective populations). Feedback populations are less specific. Excitatory neurons in one level project broadly to all excitatory neurons in the previous level. These connections are mediated exclusively through NMDA receptors (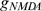 = 0.007 nS).

The router is made of two networks identical to the local modules in the sensory areas, but setting the value of 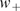 to 1.9. Each sensory modality projects to a different network in the router. Specifically, each selective population from the last sensory level projects to one selective population in the router (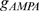 = 0.05 nS).

#### Motor network

Motor commands are simulated as in [44]. Each network in the router projects to a different motor circuit. Selective populations in the router project to one inhibitory population in the motor network (neurons from the caudate nucleus in [44]) (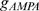 = 1.56 nS) which with enough excitation inhibit the tonic inhibition of motor neurons and enable a response. Router neurons also project to excitatory motor neurons in the motor circuit (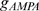 = 3.5 nS). The disynaptic inhibitory circuit by which the router both excites the different motor neurons and inhibits the tonic inhibition of those same motor units implements a threshold detection mechanism for the activity in the router. See the study of Lo & Wang [44] for a detailed description of this network.

#### Task-setting network

The task-setting network is composed of two identical modules. Each of these is composed of two populations, one excitatory (400 neurons) and one inhibitory (100 neurons). Excitatory neurons connect to themselves (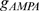 = 0.1144 nS and 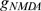 = 0.3597 nS), to the inhibitory neurons in the same module (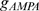 = 0.081 nS and 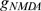 = 0.258 nS), and to the inhibitory neurons in the other module (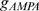 = 0.081 nS) which prohibits the simultaneous activation of both excitatory populations. Each excitatory population in the task-setting network receives input from one sensory modality, specifically from all excitatory neurons in the last level of the hierarchy (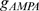 = 0.125 nS). The same sensory modality projects to one module in the router in a selective manner: each selective population in the last sensory level projects to only one selective population in the router (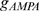 = 0.05 nS). The same router module receives excitatory input from one excitatory population in the task-setting network (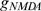 = 0.0095 nS, targeting all excitatory neurons).

#### Order-setting network

The order in which tasks are performed is controlled by an additional network (Order-setting network) which inhibits the portion of the task-setting network responsible for the amplification of the second task, until the response to the first task is emitted. The mechanism by which this occurs is as follows. The order network is a bistable network composed of one excitatory and one inhibitory population of 400 and 100 neurons respectively (self-recurrent excitatory connections: 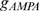 = 0.1144 nS and 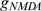 = 0.3597 nS; from excitatory to inhibitory neurons: 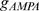 = 0.0810 nS and 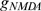 = 0.2580 nS; self-recurrent inhibitory connections: 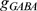 = 0.973 nS; from inhibitory to excitatory neurons: 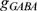 = 1.25 nS). The excitatory population in this network projects to the inhibitory population of the task-setting network which connects to the excitatory population gating the processing of the second task. A few hundred milliseconds (300 ms) before the presentation of the first task-related stimulus, excitatory neurons in the order-setting network are activated by a brief (100 ms) external input. Since the network is bistable due to the strong self-recurrent connections, it maintains high levels of activity after removal of the external input, tonically inhibiting the excitatory neurons in the task-setting network - rendering it incapable of amplifying router neurons responsible for triggering T2. When the response to T1 is emitted, a ‘corollary discharge’ from motor neurons to the inhibitory neurons in the order-setting network turns it off allowing sensory neurons from T2 to activate the task-setting network. The order network is not responsible for serial behavior in the network, since a typical PRP curve is observed even when this network is removed (Figure S2).

#### Inhibitory control and background noise

Inhibitory mechanisms were included in the network to avoid response perseveration. Direct connections were included between bursting motor neurons and local inhibitory neurons in: router (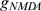 = 0.11 nS), task-setting (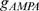 = 0.09 nS and 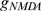 = 0.06 nS), and last sensory (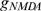 = 0.11 nS) networks. Resetting the router and the task-setting network assures a fast return to baseline activity, while the critical inhibitory signal to avoid multiple responses to the same stimulus is the one that shuts down sensory neurons. Response perseveration when these inhibitory signals are removed is shown in Figure S1.

As in previous works [31], [37], [44], [174], all neurons receive background Poisson inputs with approximate mean conductances (in nS) of: 9.9 for sensory and router excitatory neurons, 7.7 for sensory and router inhibitory neurons, 6.8 for order and task-setting excitatory neurons, 5.7 for inhibitory neurons in the task-setting network and of 5.3 for inhibitory neurons in the order network. All inputs external to the network - including background noise - are mediated by AMPA receptors.

### Stimuli

The proposed network simulates a generic PRP experiment. Observers (and the network) must perform two tasks as fast as possible, in a pre-specified order. Each task involves a simple two-alternative decision. In the network, the set of possible task-related stimuli in each modality is restricted to two, as is often the case in real PRP experiments.

All neurons receive background Poisson input to maintain a spontaneous activity of a few Hertz. The presentation of a task-relevant stimulus increased the external input of the four selective populations in the first level sensory network, from the background level of 2,400 Hz (as may result from 800 afferent neurons spiking at a spontaneous rate of 3Hz) to 2,717 Hz, for 100 ms (thus 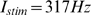). All external inputs, both background and stimulus-related, are mediated exclusively by AMPA receptors.

In Figure 4 we investigated the effect of changing the complexity of sensory processing. This was implemented by adding one additional module in the sensory hierarchy, between levels two and three. This additional module had the same number of neurons and recurrent, feedforward, and feedback parameters as the other sensory modules, with *w* = 1.94. In the same figure we also showed the effect of changing the amount of sensory evidence in favor of the correct decision. In this case, the input to the stimulus projecting to the correct response was 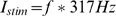 and to the other 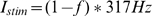 , with *f* = 0.92 in the high ambiguity case (*f* = 1 in all other simulations).

In the attentional blink (AB) simulations, a mask is presented after the task-relevant stimulus. This was modeled as in previous studies [71]. After the stimulus is removed, the external input to the non-selective cells in the first level sensory network is increased, from the background level of 2,400 Hz to 2,880 Hz, during 100 ms (thus 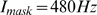).

### Simulations

Each simulated trial lasted 3400 ms. The first stimulus was presented at 700 ms, and the second stimulus was presented according to the SOA. The code was written in *C++*, and simulations were performed in the CECAR computer cluster (Buenos Aires University). Equations were integrated with the first-order Euler method, with a time step of 0.05 ms. When run on a Linux 3.16 Ghz Pentium IV PC, each trial takes about 3 minutes to complete.

## Supporting Information

Figure S1 Response perseveration without ‘corollary discharge’ from motor neurons. Smoothed firing rates of selected populations - during one trial of single-task performance - when the ‘corollary discharge’ from motor neurons to inhibitory neurons participating in memory maintenance - last level sensory neurons - is removed. Response times are indicated with red vertical arrows. (A) Stimulus selective neurons from the first sensory level show a phasic response to stimulus presentation. (B) Last level sensory areas maintain high levels of activity until a response is emitted; in the absence of inhibition from motor neurons, these neurons keep feeding routing (C) and task-setting (D) neurons, resulting in response perseveration.
(0.55 MB TIF)

Figure S2 Stochasticity in task choice. In the main simulations the order in which tasks have to be performed is constrained to mimic the condition of most PRP experiments. A recent experiment [1] investigated the decision process when the order is not specified. Results show that the proportion of trials in which participants responded first to the stimulus presented first followed a sigmoidal dependence with SOA, indicating that task order is determined by presentation order - but with a strong temporal jitter. We run a similar experiment with the proposed network. To accomplish this, we completely removed the network that controls response order (see figure 1 in main text). (A) Probability of inverting the order of the responses (i.e., responding first to the stimulus presented second) as a function of SOA. The dependence is similar to that observed experimentally [1]. (B) A typical PRP curve is observed when RTs are grouped according to the order in which responses are emitted, showing that the PRP effect does not depend on the order setting network. 1. Sigman M, Dehaene S (2006) Dynamics of the Central Bottleneck: Dual-Task and Task Uncertainty. PLoS Biol 4: e220.
(0.15 MB TIF)

Figure S3 Input currents to the router circuit during different processing stages of T2. Input currents to the router during three different processing phases of T2: before stimulus presentation (“Spontaneous”, left panel), during the phase in which T1 is being routed and S2 is buffered in memory (“Queued”, center panel), and during routing of T2 (“Routing”, right panel). The mean recurrent inputs (y-axis) flowing through AMPA (blue trace), NMDA (green trace), and GABA (red trace) receptors were obtained by simulating 50 PRP trials at SOA = 0 ms, recording these currents every 2 milliseconds. The time windows considered for each phase were (x-axis): Rest: [−150,0] ms relative to stimulus presentation; Queued: window of 150 ms centered (in each trial) around the time that the T1 task-setting neurons were active; Routing: [−150,0] ms relative to the response time to the second task. Shades depict the standard error of the mean. To assure that each of these windows overlapped with the corresponding processing stages independently of fluctuations in response time, we filtered the trials, considering only the subset of trials (37 of 50) for which the following conditions were met: T1 task-setting neurons were active for more than 150 ms and less than 350 ms, and RT2 <1000 ms.
(0.28 MB TIF)

Figure S4 Spectral analysis of sensory, router, and task-setting neurons involved in T2 processing. We analyzed the spectrogram of sensory (left panel), routing (center panel) and task setting (right panel) T2 neurons throughout the trial. Colored circles at the top identify the populations analyzed in the notation of Figure 1. The x-axis depicts the time relative to the response to the first task, and the y-axis are the frequencies (in Hz), restricted to the range 20 to 100 Hz. Data is obtained from the PRP simulation at an SOA of 0 ms. The time series of spikes from 80 neurons in each population were filtered with a Gaussian kernel (σ = 2 ms) in order to obtain the spike density function. The spectrum was estimated on 200 ms windows in sliding steps of 10 ms using 3 Slepian data tapers (windows in frequency domain) giving a frequency resolution of ±10Hz [1]. Spectrograms where calculated for each trial independently, averaged time-locked to RT1, and normalized to their maximum value to obtain the mean normalized power shown in the figure. Averages are calculated over 83 trials obtained after discarding from 100 simulations those where RT2 was higher than 800 ms. Calculations were performed with scripts from the Chronux suite (www.chronux.org). 1. Pesaran B, Pezaris JS, Sahani M, Mitra PP, Andersen RA (2002) Temporal structure in neuronal activity during working memory in macaque parietal cortex. Nat Neurosci 5: 805–811.
(0.45 MB TIF)

Figure S5 Spike-density coherence between sensory and router neurons during different processing stages. We measured the spike density coherence between sensory and router neurons, during three different phases of task processing: before stimulus presentation (left column), during active routing of T1 (center column), and during active routing of T2 (right column). Each phase lasts 100 ms. The top row corresponds to populations selective to T1 and bottom row to T2. Each population is the scheme is colored following the nomenclature of Figure 1. The coherence magnitude is shown in the y-axis, and the frequencies in the x-axis. Significant phase coherence in T1 neurons was only observed during S1 routing (top-middle panel). Significant phase coherence in T2 neurons was observed during S2 passive queuing and S2 routing. The frequency dependence of coupling during routing (top-middle panel and right-bottom panel) were similar, both showing significant coupling for low frequencies. This result may be caused by the high-frequency driving of router-neurons by the task-setting circuit. On the contrary, the coherence function during S2 queuing showed a comparable effect for low and high frequencies. Significance levels for the coherence estimates at 95% are depicted as horizontal dashed lines. Calculations were performed with the multi-taper method using the Chronux suite (www.chronux.org). Data was obtained from 200 PRP trials at a SOA of 0 ms, and the spike density function was obtained by filtering the time series of spikes from 80 neurons in each population with a Gaussian kernel (σ = 2 ms).
(0.27 MB TIF)

Figure S6 Combinatorial router. A) In the model, each neuron in the router coded exclusively for particular combinations of stimulus and response. This would lead to scaling issues when the number of possible mappings increases. Here we sketch a different coding schema for the router, one that works on the basis of combinatorial codes. A small portion of the network is simulated, containing only parts of the router and motor networks. See the main text and the model of Lo & Wang (Lo & Wang, 2006) for details of the circuit, specially the disynaptic inhibitory circuit by which the router both excites the different motor neurons and inhibit the tonic inhibition of those same motor units, implementing a threshold detection mechanism for the activity in the router. Two populations of excitatory neurons need to code for three different stimuli, each one mapped to a different response. Thus, the simultaneous activation of both router populations lead to a third response different from the one generated by each population alone, by tuning the synaptic efficacies such that the response in the center of panel A (light brown) receives a larger excitatory input than the other responses only when both router inputs are active. (BCD) Population firing rate of both router populations - during a simulation of the model - for the three possible stimuli. X-axis indicates the time relative to stimulus onset, and y-axis depicts the population firing rate calculated with an exponential causal kernel of 20 ms. The time of the motor burst is indicated by a colored vertical line, with color codes as in panel A. The simultaneous presentations of both Stim1 and Stim3 does not lead to the superimposed execution of the two responses obtained when each stimulus is presented alone (panels B and D), but to a third and different response (panel C). Thus, the implementation of inhibitory control mechanisms to arrange the sequential routing of tasks presented at short SOA is required in a combinatorial code to achieve precise stimulus-response mappings - and may lead to through the same mechanisms discussed in the main text to dual-task interference as observed in the PRP and the AB. Lo, C. C., & Wang, X. J. (2006). Cortico-basal ganglia circuit mechanism for a decision threshold in reaction time tasks. Nature Neuroscience, 9, 956–963.
(0.32 MB TIF)

Text S1 Supporting Notes.
(0.02 MB DOC)

Table S1 Results of the ANOVAs of the interference simulations. Each column corresponds to a different ANOVA. Each line represents a different effect: task manipulation, SOA, and their interaction. The top row indicates the identity of the variable under analysis and the second row indicates the type of manipulation (i.e., Notation 1 corresponds to a perceptual manipulation of the first task). Red indicates a significant effect.
(0.03 MB DOC)
