# Supplementary figures and images for: The Brain's Router: A Cortical Network Model of Serial Processing in the Primate Brain
Source: PLoS Comput Biol. 2010 Apr 29;6(4):e1000765. doi: 10.1371/journal.pcbi.1000765 (PMC2861701; doi:10.1371/journal.pcbi.1000765)

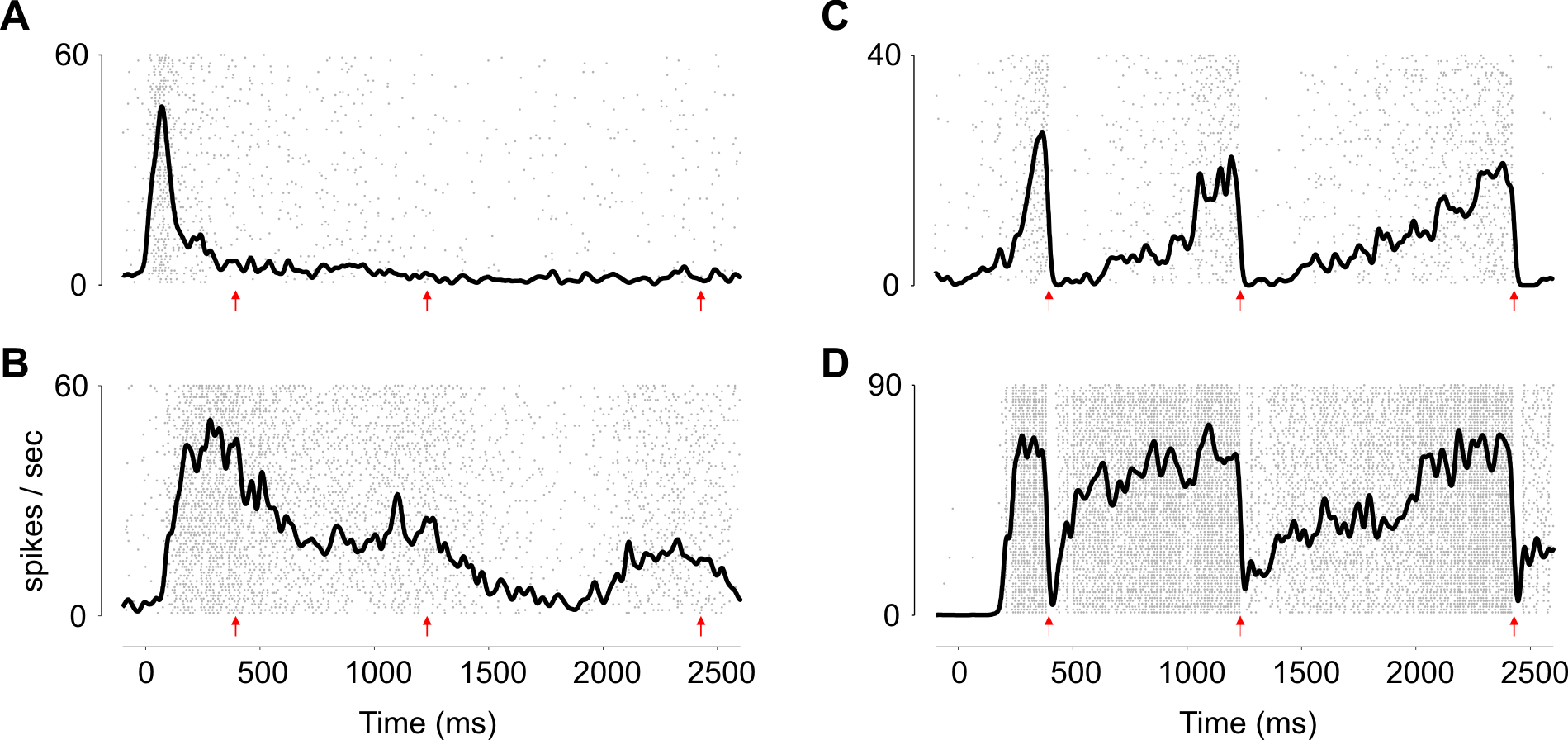

Supplement: Figure S1 — Response perseveration without ‘corollary discharge’ from motor neurons. Smoothed firing rates of selected populations - during one trial of single-task performance - when the ‘corollary discharge’ from motor neurons to inhibitory neurons participating in memory maintenance - last level sensory neurons - is removed. Response times are indicated with red vertical arrows. (A) Stimulus selective neurons from the first sensory level show a phasic response to stimulus presentation. (B) Last level sensory areas maintain high levels of activity until a response is emitted; in the absence of inhibition from motor neurons, these neurons keep feeding routing (C) and task-setting (D) neurons, resulting in response perseveration. (0.55 MB TIF) [file pcbi.1000765.s001.tif]

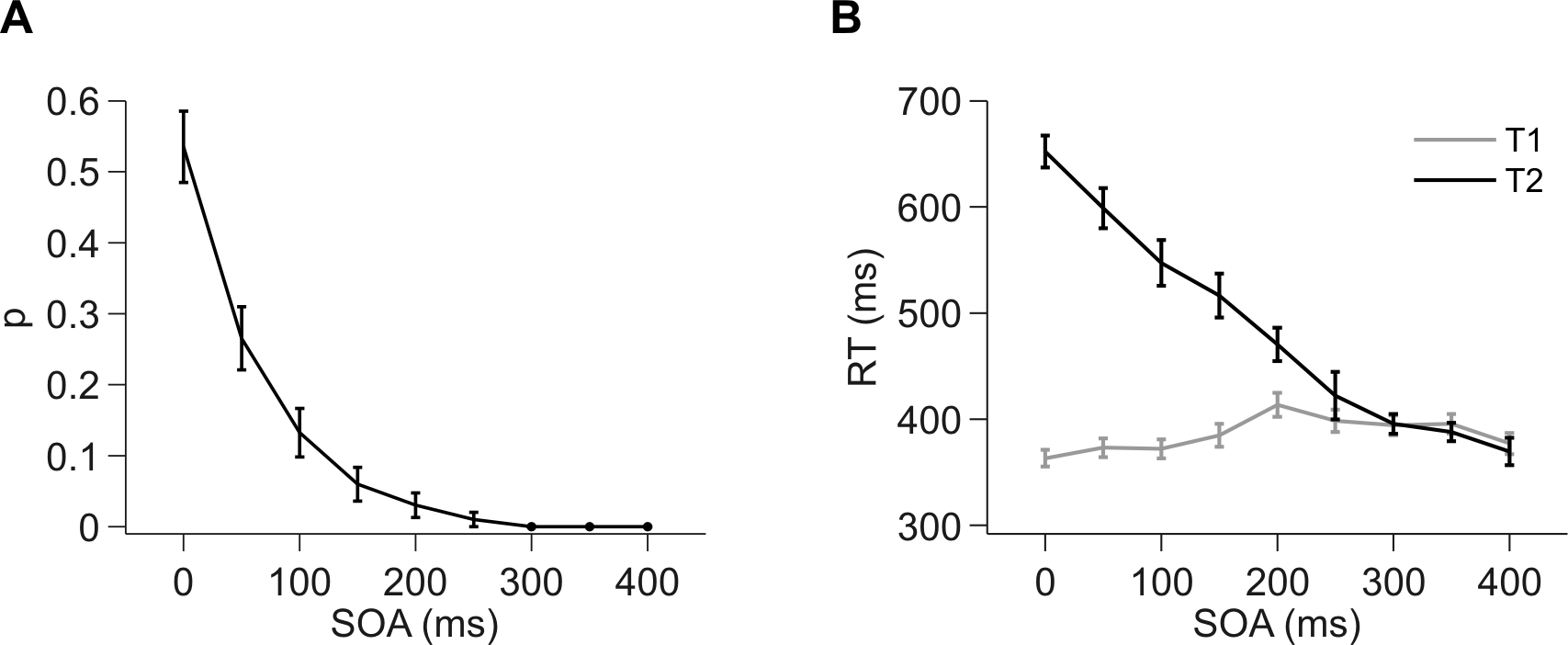

Supplement: Figure S2 — Stochasticity in task choice. In the main simulations the order in which tasks have to be performed is constrained to mimic the condition of most PRP experiments. A recent experiment [1] investigated the decision process when the order is not specified. Results show that the proportion of trials in which participants responded first to the stimulus presented first followed a sigmoidal dependence with SOA, indicating that task order is determined by presentation order - but with a strong temporal jitter. We run a similar experiment with the proposed network. To accomplish this, we completely removed the network that controls response order (see figure 1 in main text). (A) Probability of inverting the order of the responses (i.e., responding first to the stimulus presented second) as a function of SOA. The dependence is similar to that observed experimentally [1]. (B) A typical PRP curve is observed when RTs are grouped according to the order in which responses are emitted, showing that the PRP effect does not depend on the order setting network. 1. Sigman M, Dehaene S (2006) Dynamics of the Central Bottleneck: Dual-Task and Task Uncertainty. PLoS Biol 4: e220. (0.15 MB TIF) [file pcbi.1000765.s002.tif]

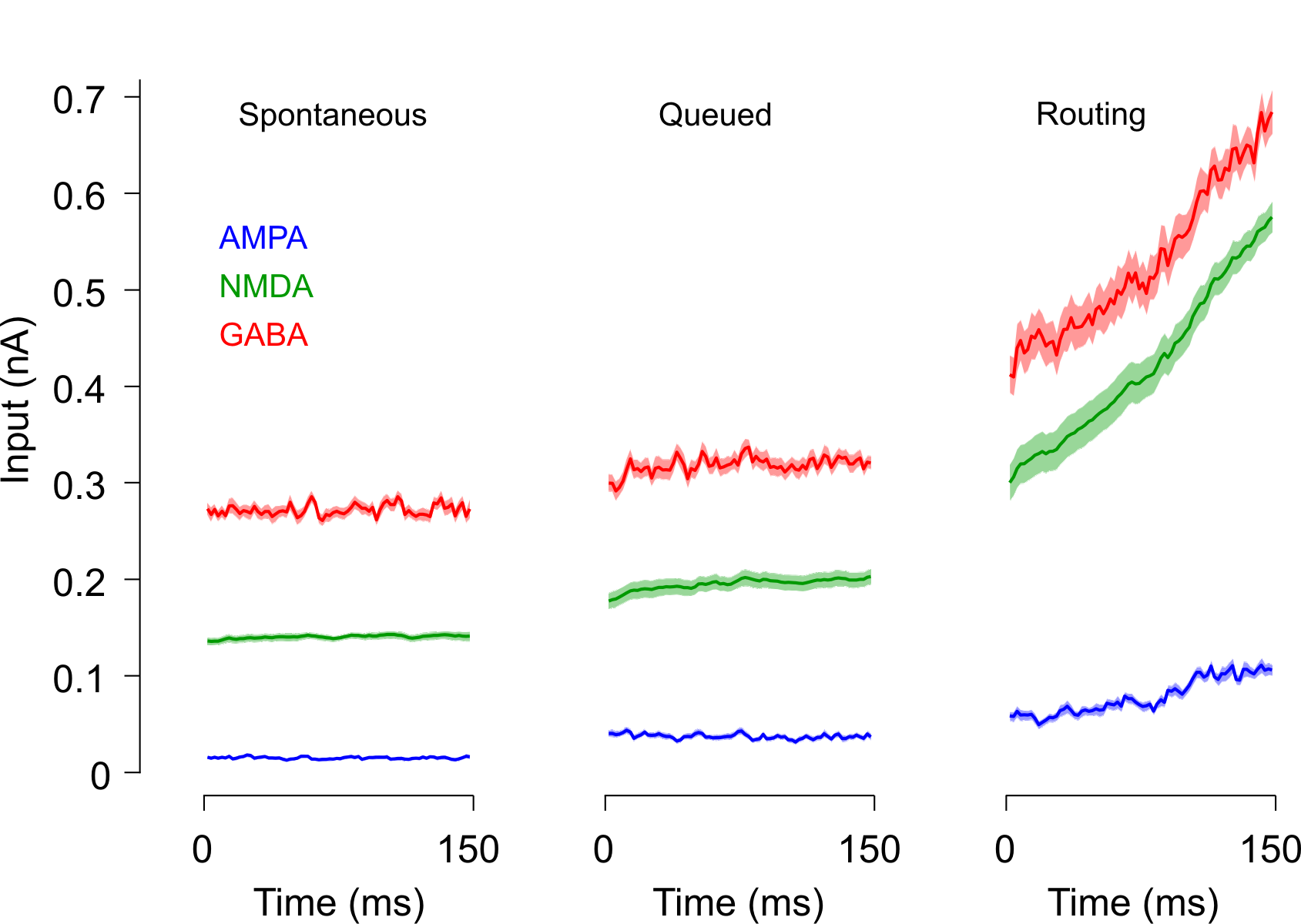

Supplement: Figure S3 — Input currents to the router circuit during different processing stages of T2. Input currents to the router during three different processing phases of T2: before stimulus presentation (“Spontaneous”, left panel), during the phase in which T1 is being routed and S2 is buffered in memory (“Queued”, center panel), and during routing of T2 (“Routing”, right panel). The mean recurrent inputs (y-axis) flowing through AMPA (blue trace), NMDA (green trace), and GABA (red trace) receptors were obtained by simulating 50 PRP trials at SOA = 0 ms, recording these currents every 2 milliseconds. The time windows considered for each phase were (x-axis): Rest: [−150,0] ms relative to stimulus presentation; Queued: window of 150 ms centered (in each trial) around the time that the T1 task-setting neurons were active; Routing: [−150,0] ms relative to the response time to the second task. Shades depict the standard error of the mean. To assure that each of these windows overlapped with the corresponding processing stages independently of fluctuations in response time, we filtered the trials, considering only the subset of trials (37 of 50) for which the following conditions were met: T1 task-setting neurons were active for more than 150 ms and less than 350 ms, and RT2 <1000 ms. (0.28 MB TIF) [file pcbi.1000765.s003.tif]

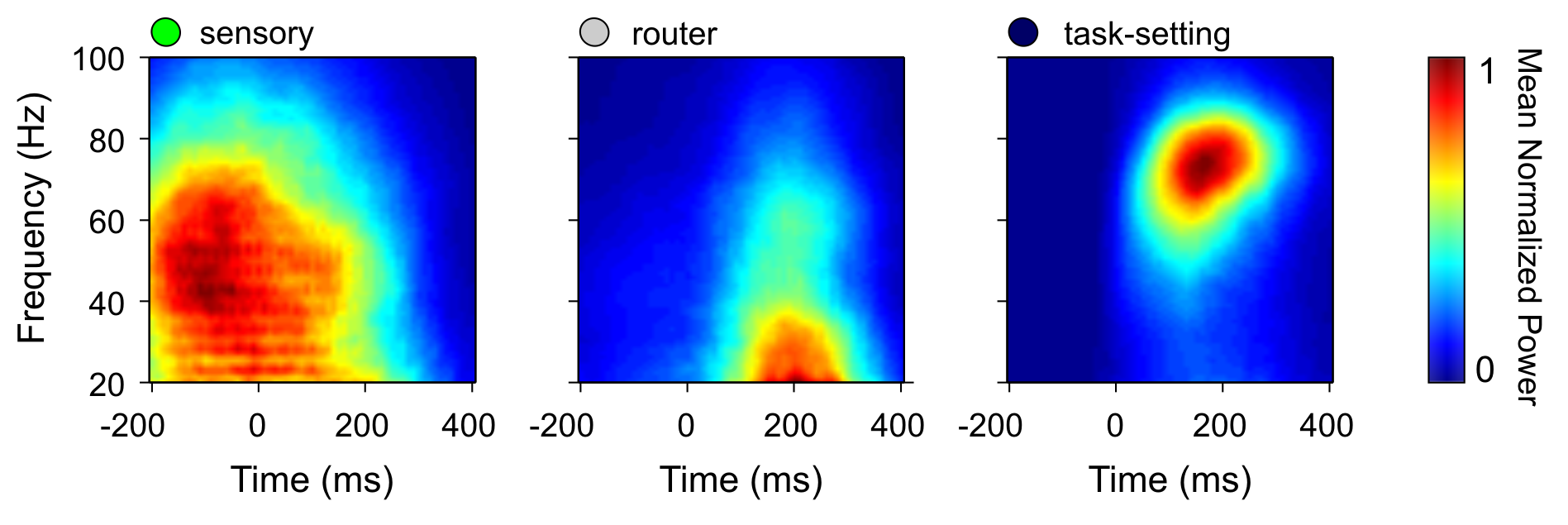

Supplement: Figure S4 — Spectral analysis of sensory, router, and task-setting neurons involved in T2 processing. We analyzed the spectrogram of sensory (left panel), routing (center panel) and task setting (right panel) T2 neurons throughout the trial. Colored circles at the top identify the populations analyzed in the notation of Figure 1. The x-axis depicts the time relative to the response to the first task, and the y-axis are the frequencies (in Hz), restricted to the range 20 to 100 Hz. Data is obtained from the PRP simulation at an SOA of 0 ms. The time series of spikes from 80 neurons in each population were filtered with a Gaussian kernel (σ = 2 ms) in order to obtain the spike density function. The spectrum was estimated on 200 ms windows in sliding steps of 10 ms using 3 Slepian data tapers (windows in frequency domain) giving a frequency resolution of ±10Hz [1]. Spectrograms where calculated for each trial independently, averaged time-locked to RT1, and normalized to their maximum value to obtain the mean normalized power shown in the figure. Averages are calculated over 83 trials obtained after discarding from 100 simulations those where RT2 was higher than 800 ms. Calculations were performed with scripts from the Chronux suite (www.chronux.org). 1. Pesaran B, Pezaris JS, Sahani M, Mitra PP, Andersen RA (2002) Temporal structure in neuronal activity during working memory in macaque parietal cortex. Nat Neurosci 5: 805–811. (0.45 MB TIF) [file pcbi.1000765.s004.tif]

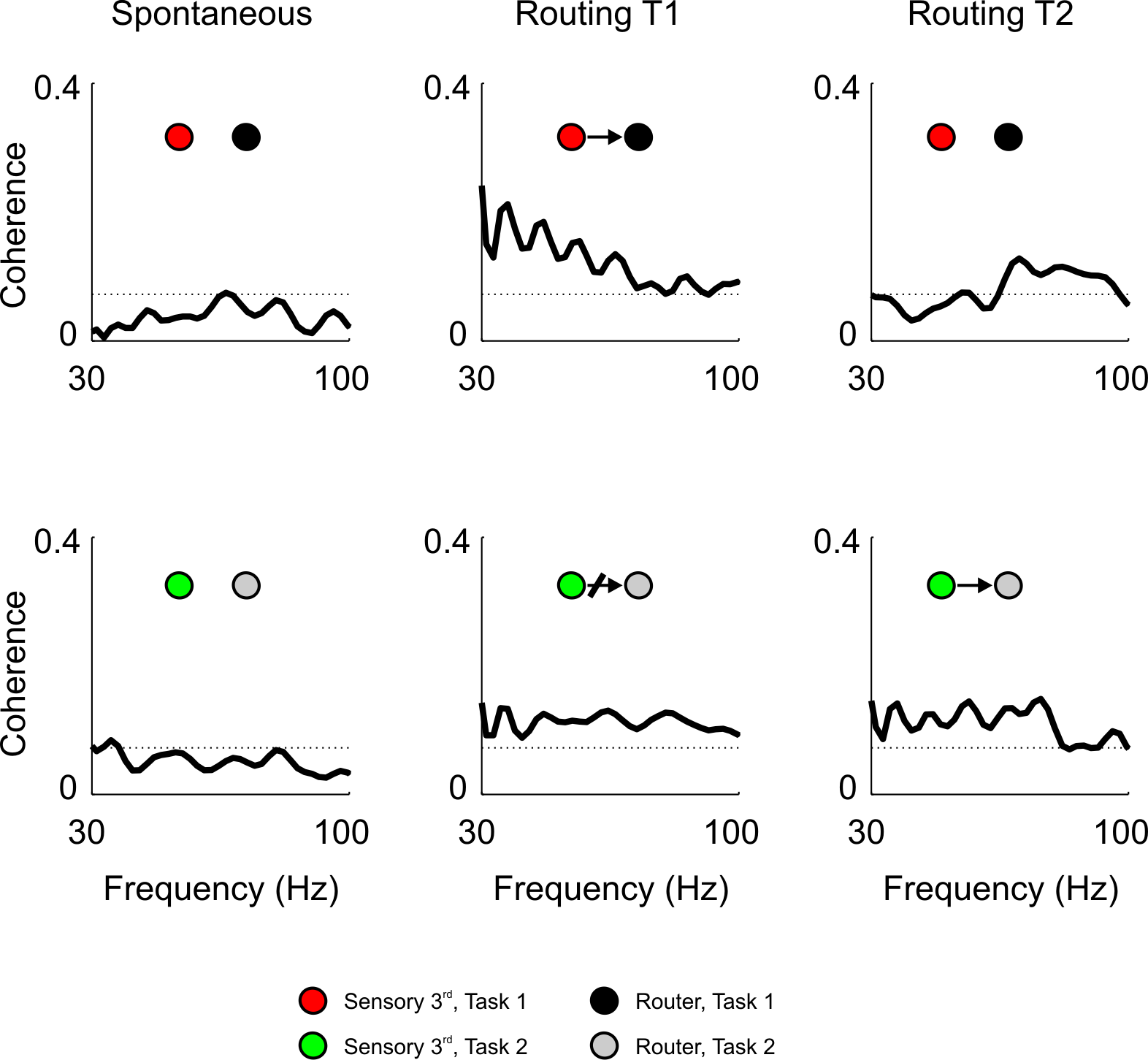

Supplement: Figure S5 — Spike-density coherence between sensory and router neurons during different processing stages. We measured the spike density coherence between sensory and router neurons, during three different phases of task processing: before stimulus presentation (left column), during active routing of T1 (center column), and during active routing of T2 (right column). Each phase lasts 100 ms. The top row corresponds to populations selective to T1 and bottom row to T2. Each population is the scheme is colored following the nomenclature of Figure 1. The coherence magnitude is shown in the y-axis, and the frequencies in the x-axis. Significant phase coherence in T1 neurons was only observed during S1 routing (top-middle panel). Significant phase coherence in T2 neurons was observed during S2 passive queuing and S2 routing. The frequency dependence of coupling during routing (top-middle panel and right-bottom panel) were similar, both showing significant coupling for low frequencies. This result may be caused by the high-frequency driving of router-neurons by the task-setting circuit. On the contrary, the coherence function during S2 queuing showed a comparable effect for low and high frequencies. Significance levels for the coherence estimates at 95% are depicted as horizontal dashed lines. Calculations were performed with the multi-taper method using the Chronux suite (www.chronux.org). Data was obtained from 200 PRP trials at a SOA of 0 ms, and the spike density function was obtained by filtering the time series of spikes from 80 neurons in each population with a Gaussian kernel (σ = 2 ms). (0.27 MB TIF) [file pcbi.1000765.s005.tif]

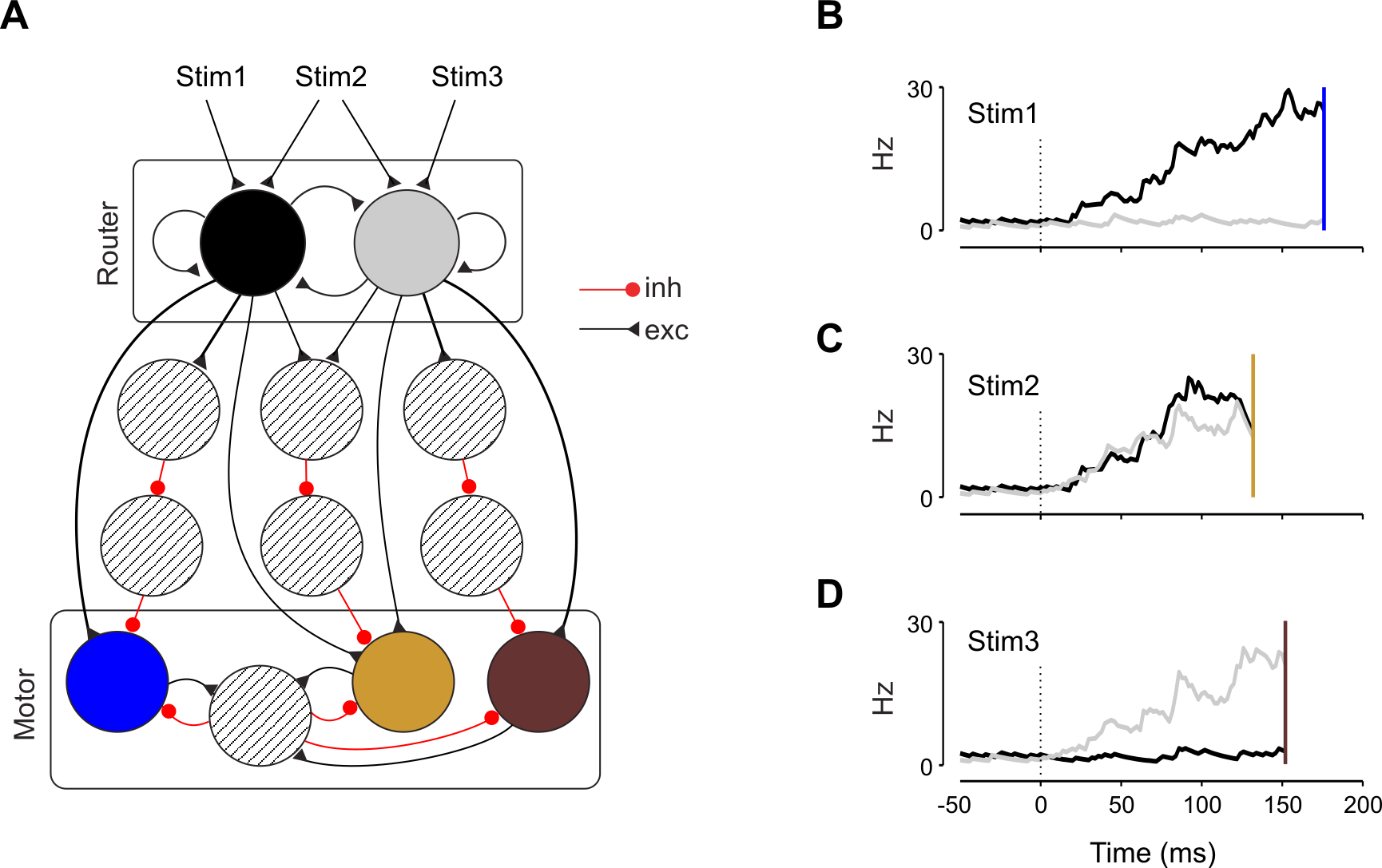

Supplement: Figure S6 — Combinatorial router. A) In the model, each neuron in the router coded exclusively for particular combinations of stimulus and response. This would lead to scaling issues when the number of possible mappings increases. Here we sketch a different coding schema for the router, one that works on the basis of combinatorial codes. A small portion of the network is simulated, containing only parts of the router and motor networks. See the main text and the model of Lo & Wang (Lo & Wang, 2006) for details of the circuit, specially the disynaptic inhibitory circuit by which the router both excites the different motor neurons and inhibit the tonic inhibition of those same motor units, implementing a threshold detection mechanism for the activity in the router. Two populations of excitatory neurons need to code for three different stimuli, each one mapped to a different response. Thus, the simultaneous activation of both router populations lead to a third response different from the one generated by each population alone, by tuning the synaptic efficacies such that the response in the center of panel A (light brown) receives a larger excitatory input than the other responses only when both router inputs are active. (BCD) Population firing rate of both router populations - during a simulation of the model - for the three possible stimuli. X-axis indicates the time relative to stimulus onset, and y-axis depicts the population firing rate calculated with an exponential causal kernel of 20 ms. The time of the motor burst is indicated by a colored vertical line, with color codes as in panel A. The simultaneous presentations of both Stim1 and Stim3 does not lead to the superimposed execution of the two responses obtained when each stimulus is presented alone (panels B and D), but to a third and different response (panel C). Thus, the implementation of inhibitory control mechanisms to arrange the sequential routing of tasks presented at short SOA is required in a combinatorial code [file pcbi.1000765.s006.tif]
