# Supplementary material for: The Brain's Router: A Cortical Network Model of Serial Processing in the Primate Brain
Source: PLoS Comput Biol. 2010 Apr 29;6(4):e1000765. doi: 10.1371/journal.pcbi.1000765 (PMC2861701; doi:10.1371/journal.pcbi.1000765)
Supplement: Text S1 — Supporting Notes. (0.02 MB DOC) [file pcbi.1000765.s007.doc]

**A**. The activity of neurons in the router which were not involved in any of the tasks explored in these experiments would have varied within a very narrow range, never reaching high level of activities. For simplicity, we did not model these neurons explicitly and thus consider their activity as part of the background noise in the router from a non-specific population.

**B**. The order network is not responsible for serial behavior in the network, since a typical PRP curve is observed even when this network is removed (see Figure S2).

**C**. Actually for short SOA values we observed a paradoxical result: the most difficult condition was processed faster. This effects results from the fact that the sensory input to the router of the more complex stimulus is stronger and thus elicits a more rapid integration. We are not aware of such a finding in PRP experiments, but word vs. letter superiority effects (higher accuracy in recognizing a letter in the context of a word than when presented in isolation or within a nonword) have often been found in the literature (for instance, see [1]).

1. Reicher GM (1969) Perceptual recognition as a function of meaningfulness of stimulus material. J Exp Psychol 81: 275-280.
