# Supplementary material for: The Brain's Router: A Cortical Network Model of Serial Processing in the Primate Brain
Source: PLoS Comput Biol. 2010 Apr 29;6(4):e1000765. doi: 10.1371/journal.pcbi.1000765 (PMC2861701; doi:10.1371/journal.pcbi.1000765)
Supplement: Table S1 — Results of the ANOVAs of the interference simulations. Each column corresponds to a different ANOVA. Each line represents a different effect: task manipulation, SOA, and their interaction. The top row indicates the identity of the variable under analysis and the second row indicates the type of manipulation (i.e., Notation 1 corresponds to a perceptual manipulation of the first task). Red indicates a significant effect. (0.03 MB DOC) [file pcbi.1000765.s008.doc]

**Table S1. Results of the ANOVAs of the interference simulations**

|  | **RT1** | | | | **RT2** | | | |
| --- | --- | --- | --- | --- | --- | --- | --- | --- |
|  | **Notation 1** | **Notation 2** | **Distance 1** | **Distance 2** | **Notation 1** | **Notation 2** | **Distance 1** | **Distance 2** |
| **Main effect of task manipulation** | p < 2.0e-16 | p = 0.1 | p < 2.2e-16 | p = 0.72 | p < 2.2e-16 | p < 2.2e-16 | p < 2.2e-16 | p < 2.0e-16 |
|  | F = 911.8 | F = 2.6 | F = 736 | F = 297 | F = 165 | F = 79 | F = 191 | F = 297 |
| **Main effect of SOA** | p = 0.82 | p = 0.69 | p = 0.98 | p = 0.90 | p < 2.2e-16 | p < 2.2e-16 | p < 2.2e-16 | p < 2.0e-16 |
|  | F = 0.6079 | F = 0.74 | F = 0.29 | F = 231 | F = 379 | F = 245 | F = 297 | F = 231 |
| **Interaction** | p = 0.99 | p = 0.76 | p = 0.35 | p = 0.99 | p < 2.2e-16 | p < 2.2e-16 | p < 2.2e-16 | p = 0.735 |
|  | F = 0.23 | F = 0.67 | F = 1.1 | F = 0.7 | F = 13 | F = 16 | F = 11 | F = 0.7049 |

Each column corresponds to a different ANOVA. Each line represents a different effect: task manipulation, SOA, and their interaction. The top row indicates the identity of the variable under analysis and the second row indicates the type of manipulation (i.e., Notation 1 corresponds to a perceptual manipulation of the first task). Red indicates a significant effect.
